# Specvis: Free and open-source software for visual field examination

**DOI:** 10.1371/journal.pone.0186224

**Published:** 2017-10-13

**Authors:** Piotr Dzwiniel, Mateusz Gola, Anna Wójcik-Gryciuk, Wioletta J. Waleszczyk

**Affiliations:** 1 Nencki Institute of Experimental Biology of the Polish Academy of Sciences, Warsaw, Poland; 2 Institute of Psychology of the Polish Academy of Sciences, Warsaw, Poland; 3 Swartz Center for Computational Neuroscience, Institute for Neural Computation, University of California, San Diego, California, United States of America; 4 Mediq Clinic, Legionowo, Poland; Bascom Palmer Eye Institute, UNITED STATES

## Abstract

Visual field impairment affects more than 100 million people globally. However, due to the lack of the access to appropriate ophthalmic healthcare in undeveloped regions as a result of associated costs and expertise this number may be an underestimate. Improved access to affordable diagnostic software designed for visual field examination could slow the progression of diseases, such as glaucoma, allowing for early diagnosis and intervention. We have developed Specvis, a free and open-source application written in Java programming language that can run on any personal computer to meet this requirement (http://www.specvis.pl/). Specvis was tested on glaucomatous, retinitis pigmentosa and stroke patients and the results were compared to results using the Medmont M700 Automated Static Perimeter. The application was also tested for inter-test intrapersonal variability. The results from both validation studies indicated low inter-test intrapersonal variability, and suitable reliability for a fast and simple assessment of visual field impairment. Specvis easily identifies visual field areas of zero sensitivity and allows for evaluation of its levels throughout the visual field. Thus, Specvis is a new, reliable application that can be successfully used for visual field examination and can fill the gap between confrontation and perimetry tests. The main advantages of Specvis over existing methods are its availability (free), affordability (runs on any personal computer), and reliability (comparable to high-cost solutions).

## Introduction

The main causes of human visual field impairment include cataracts, glaucoma, age related macular degeneration, diabetic retinopathy, and stroke (for review see [1–8]). These conditions may lead permanently to partial and/or complete blindness. Over 100 million people worldwide suffer from these impairments and this number increases yearly [9–14]. Although limited reversal of visual impairment may be possible in some cases [15–21], in general, full restoration is currently not possible. Due to limited access to ophthalmic healthcare and its associated costs, especially in developing countries where existing visual field examination methods are rudimentary, it’s highly likely that the number of future patients has been underestimated. Lack of easily accessible and well equipped ophthalmic healthcare is a primary factor for late determining existing visual deficits since many people are not aware of the disease progression until this interferes with their daily life. Thus, the early diagnosis and treatment of ophthalmic disease is essential to the long-term maintenance of ophthalmic health.

According to Elliot and colleagues [22] a typical confrontation test “must involve the examiner comparing his or her visual field with that of the patient”. In practice, the examiner asks the patient to close one eye and fixate on the examiner’s nose while moving his finger from the fixation point to a region where the patient no longer reports seeing the finger and compares this to his own response. Due to its simplicity confrontation testing can be extremely useful as a fast and cheap screening method. It can be performed anywhere by support staff, however this method does not provide detailed information about the patient’s visual field.

Various types of perimetry provide a more sophisticated way for visual field examination and, in general, can be defined as a method for the dynamic assessment of sensitivity to light across the visual field [23–25]. Light stimuli are presented on a hemispherical surface with a predefined background illumination, and the examined eye is positioned at the geometric center of the hemisphere so that all surface locations are equidistant from the eye. In automated static perimetry (ASP), the patient responds to short duration stimuli statically displayed at predefined locations. ASP is a commonly used visual field examination in developed countries as a method of early detection and prevention. Two very popular devices based on the ASP’s principles are the Humphrey Field Analyzer and Medmont M700 Automated Static Perimeter (MM700). Both are very useful tools in the diagnosis of the early stages of ophthalmic disease but the associated costs are a financial burden in underdeveloped countries.

One possible way to reduce the cost of early intervention is to take advantage of the low cost and accessibility of personal computers and provide software dedicated to visual field examination. An additional advantage of such a solution is mobility, i.e. the test can be conducted almost anywhere.

The extensive list of currently available programming libraries and standalone applications for widely understood psychophysics, including visual field testing, is available at http://www.hans.strasburger.de/psy_soft.html. We have found only two applications dedicated primarily for visual field examination. These are “Visual field screening test for glaucoma” and “EyesCream Visual Field Analyzer”. However, none of these allows for accurate visual field examination comparable with commonly used perimeters.

We have also found that NovaVision Inc. (http://www.novavision.com/) does share a functionality that allows users to perform a very basic screening test online. The patient fixates on a central point and uses the keyboard to respond to white dots statically displayed (1-time only) at predefined locations on a black background. Unfortunately, there is no direct access to information about any of the examination parameters (e.g. stimulus duration or inter-stimulus interval), thus it is not possible to modify any of these. Additionally, the user must provide personal and contact information in order to perform the test.

There is also a solution called Ceeable Visual Field Analyzer (CVFA) provided by Ceeable Inc. (http://www.ceeable.com/), which is (according to its authors) a visual field test that can detect, classify, and monitor degenerative eye disease using only a tablet. This solution is not free and requires an internet connection.

Here we describe Specvis—developed by our group free and open-source application for visual field examination written in Java programming language that can run on any personal computer. While designing Specvis we aimed to meet three major requirements for our software with regard to filling the gap between confrontation and perimetry tests. Firstly, we wanted to provide free and open-source program. Secondly, the software must have been able to examine the patient’s full visual field, sensitivity to light of different wavelength and luminance, and should have been accurate and reliable for the diagnosis of visual field impairments. Lastly, software must have been user friendly enough to allow anyone using it without an intensive training. We believe there is a high need for such software, as early diagnosis of such diseases as glaucoma or retinitis pigmentosa are crucial to provide proper treatment and prevent further progression of visual impairment [26]. The capacity for accurate early diagnosis is the crucial factor, lacking often in underdeveloped countries.

## Specvis definition and usage

Specvis is freely available as an open-source application based on GNU GPLv3 license and can be run on any personal computer by anyone (does not demand any special skills). It has dedicated website (http://www.specvis.pl/) with link to GitHub repository containing application source code and executables (https://github.com/piotrdzwiniel/Specvis) as well as data from both validation studies described below (https://github.com/piotrdzwiniel/Specvis/tree/master/Additional-Data-For-PLoS). Similar to other ASPs, Specvis displays a single, specific, light stimulus at different locations on the computer screen, in order to assess a luminance threshold across the visual field. A comprehensive description of the application is given in the section ‘Software implementation’.

## Visual field examination of patients with diagnosed ophthalmic deficits

We compared the results of visual field examination of four patients with glaucoma (all females; 59.3 ± 3.3 years), one patient with retinitis pigmentosa (42 years old female) and two ischemic stroke patients with homonymous right hemianopia (both males in age of 70 and 64) after testing with the MM700 and the Specvis application. We enrolled only healthy patients (aside of diagnosed and described ophthalmic conditions) who did not show deficits in the center of the visual field and have normal or corrected to normal visual acuity (Table 1). Patients provided written informed consent concerning participation in the study. The study adhered to the Declaration of Helsinki and was approved by the Ethical Committee of the Institute of Psychology of the Polish Academy of Sciences.

**Table 1:** Patients’ general information. Visual acuity of glaucomatous (GM) patients were tested with the use of Snellen charts (5 m for best corrected visual acuity/ uncorrected visual acuity (BCVA/UVA) and 30 cm for corrected near visual acuity/ uncorrected near visual acuity (CNVA/UNVA)) at the Mega-Lens Specialized Ophthalmology Clinic in Warsaw. The intra-ocular pressure (IOP) of glaucomatous patients was also measured in the clinic. Visual acuity of retinitis pigmentosa (RP) patient was tested at the Nencki Institute of Experimental Biology of the Polish Academy of Sciences in Warsaw with the use of Early Treatment Diabetic Retinopathy Study (ETDRS) charts (5 m for BCVA/UVA and 33 cm for CNVA/UNVA). Visual acuity results are expressed in Visus scale (also known as Snellen scale).

Group	Subject	Age	Eye	BCVA	UVA	CNVA	UNVA	IOP	Time of diagnose
**GM**	1	61	Left	0.8	N/A	0.5	N/A	16	2004
			Right	0.8	N/A	0.5	N/A	16	
	2	65	Left	1.0	N/A	0.5	N/A	21	2010
			Right	1.0	N/A	0.5	N/A	13	
	3	61	Left	1.0	N/A	0.5	N/A	18	2006
			Right	1.0	N/A	0.5	N/A	20	
	4	55	Left	1.0	N/A	0.5	N/A	15	2008
			Right	1.0	N/A	0.5	N/A	16	
**RP**	1	42	Left	N/A	0.5	N/A	0.4	N/A	2006
			Right	N/A	0.6	N/A	0.5	N/A	
**ST**	1	70	Left	N/A	N/A	N/A	N/A	N/A	2017
			Right	N/A	N/A	N/A	N/A	N/A	
	2	64	Left	N/A	N/A	N/A	N/A	N/A	2017
			Right	N/A	N/A	N/A	N/A	N/A	
**Average**		59.7							
**SD**		9.1							

ST—stroke; IOP—intra-ocular pressure in mm Hg; Time of diagnose refers to first time when ophthalmic deficit was diagnosed.

### MM700 visual field examination

Visual field examinations using MM700 were performed at the Mega-Lens Specialized Ophthalmology Clinic in Warsaw in case of patients with glaucoma and at the OPTIMUM Ophthalmic Center in Warsaw in case of retinitis pigmentosa and stroke patients. Glaucomatous patients were tested with a Glaucoma Threshold Test (GTT) which examined the patient’s central 22° visual field and 50° nasally, whereas retinitis pigmentosa and stroke patients were tested with a Central Test (CT), which examined the patient’s central 30° visual field. Tests were performed using rear projection green light emitting diodes (LEDs; pale green (565 nm) spots of 0.43° equal to Goldmann III standard) presented at 104 predefined locations in case of GTT and in 100 locations in case of CT. Background luminance was equal to 3.2 cd/m^2^. The minimum and maximum stimulus luminance was equal to 3.2 and 321.2 cd/m^2^ respectively (i.e. a differential luminance spread from 0.01 to 318 cd/m^2^, or conversely a decibel range from 0 to 45.02 dB). The stimulus display time was set to 200 ms and the minimum inter-stimuli interval was 400 ms. Checking the patient’s fixation was performed using the Heijl-Krakau method [27] with an assumed blind spot location at 15° temporal and 3° inferior to the fixation point. Fixation monitoring for retinitis pigmentosa and stroke patients was performed with additional threshold stimulus in the location of fixation point, to which patient should respond. The visual field examination was performed in a darkened room and patients wore their glasses for near vision correction.

### Specvis visual field examination

**Fig 1:**
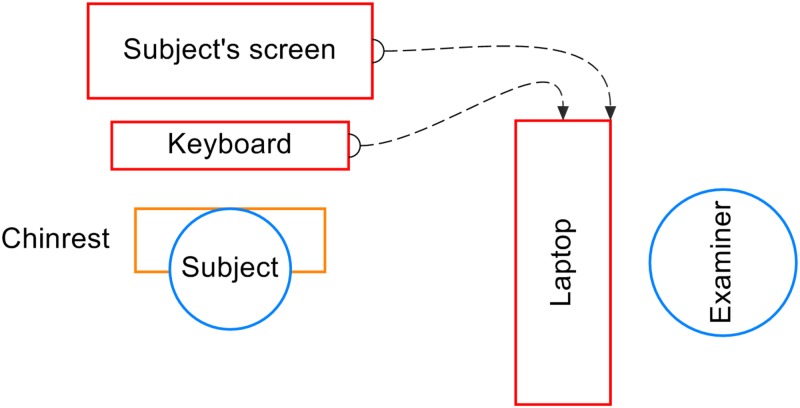
Specvis hardware configuration used in the validation. Specvis was run on a laptop (MSI G60, CPU i7 4700MQ, RAM 8GB, OS WIN 10) with an additional screen (Iiyama ProLite E2483HS, set resolution 1920 x 1080, screen width 535 mm, screen height 300 mm) viewed by the subject and keyboard (Microsoft Wired 600) for subject responses. The examiner monitored the progress of the visual field examination on the laptop via the procedural window (for more details read section ‘Conducting visual field test and monitoring test progress’). The distance between the patient and screen was 370 mm, the position and stability of the head was maintained by a chinrest.

Visual field examination using Specvis was performed within the Nencki Institute of Experimental Biology of the Polish Academy of Sciences in Warsaw. Three glaucomatous patients were tested with slightly different settings compared to a glaucomatous patient no. 4, retinitis pigmentosa and stroke patients due to the on-going Specvis development. First three glaucomatous patients were tested with Specvis prototype version not-available to public. Glaucomatous patient no. 4 was examined with the use of Specvis version 1.0, whereas retinitis pigmentosa and stroke patients with version 1.1 (both available to public on GitHub). All patients were tested with the hardware configuration shown in Fig 1. Examination was conducted in a darkened room. One eye was occluded by an elastic eye patch and the patients wore their glasses to correct their near vision.

Specvis settings for the first three glaucomatous patients were as follows. The tested visual field was 71.73° horizontally and 44.41° vertically. The luminance scale used for both the stimuli and for the background was created using the green-like color expressed on the HSB space (equal to 534 nm) with hue and saturation equal to 100. The wavelength conversion to the HSB space color was done using the “Wavelength to RGB and HEX Calculator” (http://lsrtools.1apps.com/wavetorgb/), which is based on the algorithm described at http://www.physics.sfasu.edu/astro/color/spectra.html. The scale configuration was performed with a Konica Minolta LS-100 photometer in a darkened room with the screen brightness set to 100% and contrast set to 70%. The minimum and maximum luminance values for the stimulus and background were 0.42 and 155.1 cd/m^2^ respectively. The quality of fit of the scale was characterized by a chi-squared equal to 1.63 (p = 0.898, SD = 2.41 cd/m^2^). The background fitted luminance was set to 3.13 cd/m^2^ (HSB brightness 12%). The minimum and maximum stimulus fitted luminance was equal to 3.66 (HSB brightness 12%) and 157.45 cd/m^2^ (HSB brightness 100%), respectively, with a dB range of 0 to 16.34. The stimulus had an ellipse shape with a width and height equal to 0.4°. Display time was set to 200 ms and inter-stimulus interval randomly varied between 1000 to 1500 ms. The distance between neighboring stimuli was equal to 5.98° horizontally and 5.52° vertically (96 predefined locations). The correction for the sphericity of the field of view was not available in the application at the time of testing first three glaucomatous patients. The fixation point was positioned centrally on the screen with a color expressed in the HSB space by hue and saturation equal to 0, brightness to 50, and a luminance equal to 65.7 cd/m^2^. Its shape was an ellipse with a width and height equal to 0.5°. The patients’ gaze on the fixation point was controlled with the use of Specvis technique called *Blindspot*. In short, this technique is similar to the Heijl-Krakau technique where in assumed blind spot location Specvis displays light stimulus called *control* stimulus, which by definition should not be perceived by the patient. The assumed blind spot location was set to 3° below the fixation point and 15° to the left for the left eye, and 15° to the right for the right eye (for more detailed information about this technique read section ‘Software implementation‘). The fixation control frequency was set to one fixation control for each 1 to 10 ordinary stimuli displayed. The control stimulus shape and size were the same as the ordinary stimuli (luminance = 157.45 cd/m^2^; HSB brightness 100%). The option to show fixation feedback messages for the patient was not available in this version of Specvis. The chosen procedure type was *Basic* (for more detailed information about this procedure read section ‘Software implementation‘). The brightness vector length was equal to 17 and values were spread equally. The participant responded to the stimuli by pressing the SPACE bar on the keyboard.

Thanks to the possibilities offered by Specvis in version 1.0 and 1.1 we made minor adjustments to the setting for the glaucomatous patient no. 4, as well as for the retinitis pigmentosa and stroke patients. Changes introduced for the glaucomatous patient were following: the minimum and maximum luminance values for the stimulus and background were equal to 0.54 and 153.5 cd/m^2^, respectively. The quality of the scale fit was expressed by a chi-squared equal to 2.12 (p = 0.832, SD = 1.07 cd/m^2^). The background fitted luminance was set to 3.15 cd/m^2^ (HSB brightness 10%) and the minimum and maximum stimulus fitted luminance was equal to 3.64 (HSB brightness 11%) and 154.46 cd/m^2^ (HSB brightness 100%) respectively (dB range 0 to 16.28). Inter-stimulus interval was randomly varied between 900 and 1300 ms and the distance between stimuli equal to 6° horizontally and vertically (96 predefined locations). The correction for the sphericity of the field of view was used (read section ‘Stimulus and background options’ for more detailed information about this correction). The fixation point location was moved 14° to the left from center of the screen during examination of the left eye and 14° to the right for the right eye. The fixation control frequency was set to one fixation control for each 5 to 10 stimuli displayed. The luminance of the control stimulus was equal to 154.46 cd/m^2^ (HSB brightness 100%). The option to show feedback messages to the patient about fixation loss was used. The brightness vector length in the *Basic* procedure was 13 and the values spread equally.

For retinitis pigmentosa and stroke patients, in comparison to the settings used for glaucomatous patient no. 4, we have changed inter-stimulus interval which randomly varied between 1200 and 1600 ms. The brightness vector length in the *Basic* procedure was 9 with equally spread values. Finally, we have moved the fixation point to the center of testing visual field and changed fixation monitor technique to *Both*, which use both fixation monitor techniques, *Blindspot* and *Fixation point change*, simultaneously to control patient’s fixation.

### Results

We compared Specvis and MM700 mainly in the domain of graphical representation of the patients’ visual field because both methods have different minimum and maximum luminance values for the stimuli and background, resulting in different dB ranges. The Specvis stimulus maximum luminance was 157.45 cd/m^2^ for the first three glaucomatous patients and 154.46 cd/m^2^ for the forth glaucomatous patient and for retinitis pigmentosa and stroke patients, while for all MM700 patients stimulus luminance was 321.2 cd/m^2^. A lack of response to maximum luminance stimuli in Specvis was expressed by 0 dB, whereas a luminance equal to 154.46 cd/m^2^ in MM700 is equivalent to 3.14 dB. The minimum stimulus luminance used for the glaucomatous patient no. 4 and for retinitis pigmentosa and stroke patients with Specvis was equal to 3.64 cd/m^2^ or 16.28 dB, where the same dB value in MM700 corresponds to 7.49 cd/m^2^.

Additionally, in the case of glaucomatous patients, in MM700 the density of stimulus locations in the first 10° from the fixation point was higher than in more distant areas and stimuli locations were radially arranged from the fixation point. By comparison, all stimulus locations in Specvis were spread equally and are arranged in a matrix-like fashion.

Given the above, we believe that the most appropriate comparison between MM700 and Specvis should be based only on basic data, such as test duration, and the graphical maps of visual sensitivity to visual stimuli across the visual field. Data for glaucomatous, retinitis pigmentosa and stroke patients are summarized in Table 2, S1 and S2 Tables.

**Table 2:** Summary data for the visual field examination tests for glaucomatous (GM), retinitis pigmentosa (RP) and stroke (ST) patients examined with MM700 and Specvis.

Group	Subject	Eye	MM700			Specvis		
			Duration	FA	FPRR	Duration	FA	FPRR
**GM**	1	Left	09:45.0	36/37 (97)	1/33 (3)	12:19.0	70/78 (90)	N/A
		Right	09:46.0	34/37 (92)	4/32 (13)	13:46.0	90/92 (98)	N/A
	2	Left	13:40.0	52/58 (90)	0/52 (0)	12:14.0	73/75 (97)	N/A
		Right	12:43.0	53/53 (100)	0/48 (0)	15:01.0	93/100 (93)	N/A
	3	Left	16:00.0	61/65 (94)	1/59 (2)	13:44.0	50/103 (49)	N/A
		Right	15:06.0	56/56 (100)	0/52 (0)	13:23.0	75/90 (83)	N/A
	4	Left	09:26.0	34/39 (87)	1/32 (3)	08:15.0	44/48 (92)	1/180 (1)
		Right	08:23.0	32/32 (100)	0/29 (0)	09:31.0	41/47 (87)	3/221 (1)
RP*	1	Left	10:03.0	41/41 (100)	0/38 (0)	09:02.0	39/39 (100)	0/49 (0)
		Right	09:14.0	39/39 (100)	0/35 (0)	09:05.0	39/39 (100)	0/48 (0)
ST*	1	Left	06:32.0	23/28 (82)	1/23 (4)	10:15.3	23/23 (100)	1/154 (1)
		Right	06:45.0	27/27 (100)	0/25 (0)	09:57.7	22/22 (100)	2/141 (1)
	2	Left	07:57.0	23/29 (79)	0/24 (0)	10:25.0	16/22 (72)	51/171 (18)
		Right	06:32.0	27/28 (96)	1/24 (4)	10:10.3	21/22 (95)	49/161 (22)
**Average**			10:08.0	38.4/40.6 (94.1)	0.6/36.1 (2.1)	11:13.4	49.7/57.1 (89.7)	13.4/140.6 (5.5)
**SD**			03:06.1	12.1/12.0 (6.9)	1.0/11.5 (3.4)	02:08.3	25.2/30.1 (13.7)	21.2/57.6 (8.4)

Duration—total duration of the visual field examination test; FA—fixation accuracy expressed as the number of correct responses to fixation checks/ the total number of fixation checks (%); FPRR—false-positive response rate expressed as the number of false-positive responses/ the number of positive responses (%);

*—Specvis results for retinitis pigmentosa and stroke patients averaged over three visual field examinations.

#### Glaucomatous patients

The average test duration for the initial three glaucomatous patients tested with Specvis was 34 s longer (12:50 vs. 13:24) largely due to using a longer brightness vector in *Basic* procedure (equal to 17). Reducing vector length from 17 to 13, as in the case of the glaucomatous patient no. 4, reduced the test duration by 4:31 min resulting in similar average test duration as in MM700 (8:53 vs. 8:54). However, Specvis test duration can vary widely from test to test as result of self-paced breaks introduced by the patient.

The average fixation accuracy rate in case of glaucomatous patients was lower in Specvis compared to MM700, 67/79 (85%) vs. 45/47 (96%) respectively, largely due to the very poor fixation of the glaucomatous patient’s no. 3 left eye (50/103–49%). Note also that only the glaucomatous patient no. 4 tested with Specvis was given automated feedback messages about fixation loss. In addition, only this patient had a monitored false-positive response rate (FPRR), which was somewhat better in testing with Specvis, than in MM700 (1% vs. 1.5%).

We examined and graphically mapped the central visual field (~72° horizontal and ~44° vertical) of the initial three glaucomatous patients tested with Specvis (S1–S3 Figs) vs. results obtained with the MM700 (central 22° and 50° nasal from the fixation point). Specvis allowed easy identification of the blind spot and subjective similarities in the central visual field condition compared to MM700 test results. Of note were the Specvis test results for the left eye of the glaucomatous patient no. 2 (S2 Fig). The patient reported a lack of vision in the upper regions of the visual field that was subsequently confirmed by the simpler confrontation test to a manually presented white dot. Interestingly, MM700 results for this patient suggest that patient has only slightly worse vision in this region.

**Fig 2:**
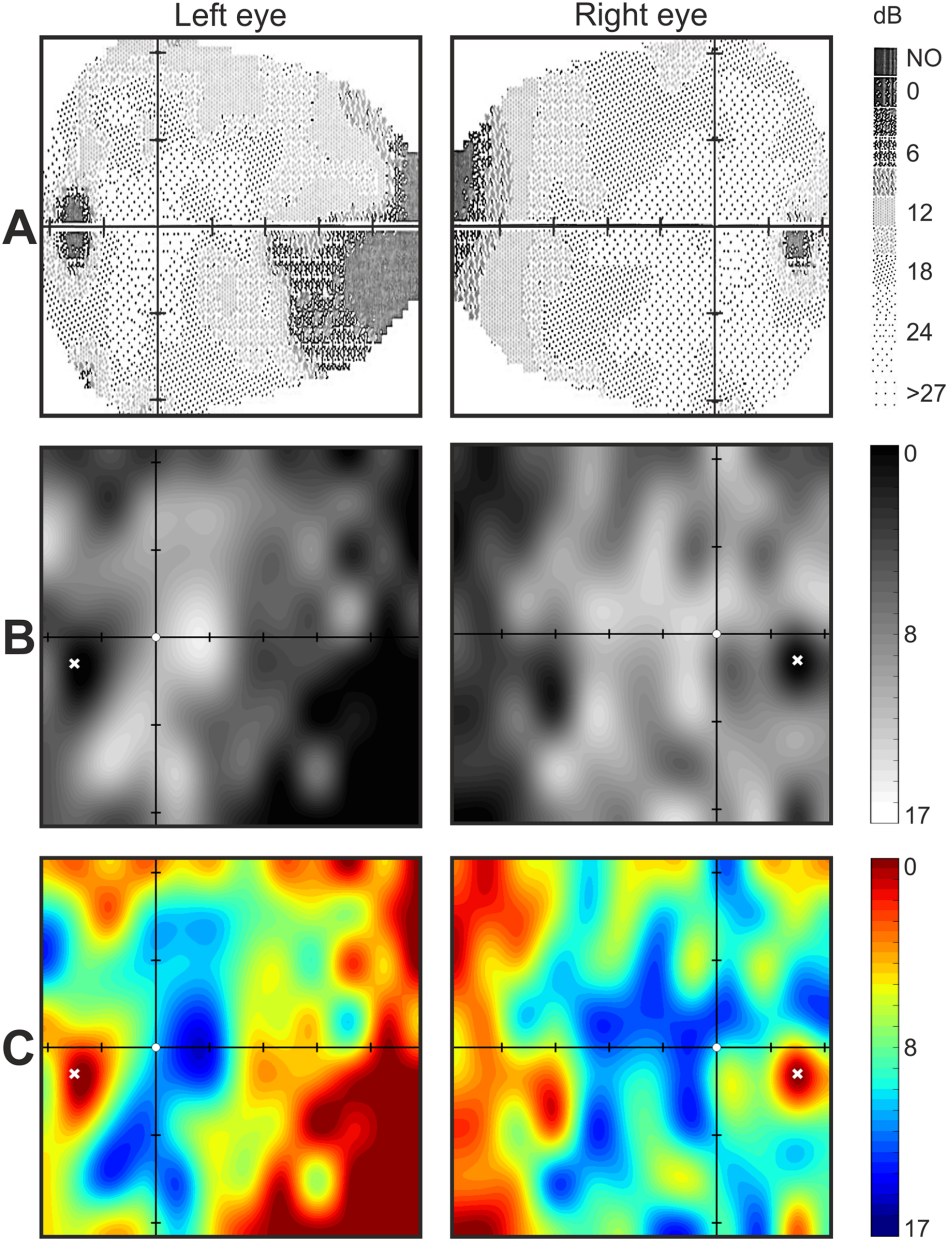
Glaucomatous patient no. 4; Medmont M700 (MM700) and Specvis visual field graphical maps. **A**. The results from MM700 were mapped according a decibel scale (dB) where NO indicates a lack of response to the stimulus in predefined locations. **B and C**. Visual field sensitivity obtained with the Specvis application shown as gray scale or color scale graphical maps in dB for easy comparison to the MM700 maps. The white X marker indicates the location for fixation control testing and therefore also represents the location of the optic disc. Axes intersect at the fixation point with tick marks at 10° intervals.

The glaucomatous patient no. 4 was tested with Specvis in version 1.0 (Fig 2), which included a correction for sphericity of the field of view and automated feedback messages about fixation loss. Additionally, during the Specvis test, we changed the fixation point location so that the whole examined visual field area encompassed the same area examined with the MM700. The location of the optic disc on the Specvis visual field graphical maps were clearly discernible and the expanded visual field representation allowed identification of a glaucomatous deficit in the left eye starting ~10° nasally (Fig 2B and 2C). This deficit was also visible on the MM700 maps (Fig 2A). Examination of the right eye revealed decreased sensitivity in the nassal visual field from ~30° when using either method.

#### Retinitis pigmentosa patient

Retinitis pigmentosa patient was tested once with MM700 for both eyes, and three times for both eyes with Specvis. Average test duration with MM700 was 9:38.5 ± 34.6 s, whereas for tests conducted with Specvis it was 9:03.3 ± 3.6 s. For retinitis pigmentosa and stroke patients we have reduced brightness vector length in *Basic* procedure to 9, which resulted in even shorter test durations than in the case of glaucomatous patients. But we have also increase inter-stimuli interval to 1200–1600 ms, which resulted in longer test durations than for glaucomatous patient no. 4.

The average fixation accuracy was great in both, MM700 and Specvis, i.e. 100.0% ± 0.0% and 99.6% ± 0.6%. For retinitis pigmentosa patient we have used fixation monitor technique *Both*, which consists of displaying *control* stimulus in predefined blind spot location, as well as changing characteristics of the fixation point, to which patient is obligated to respond. In short, technique *Both* links *Blindspot* and *Fixation point change* fixation monitor techniques. The FPRR was equal to 0.0% in both, MM700 and Specvis.

**Fig 3:**
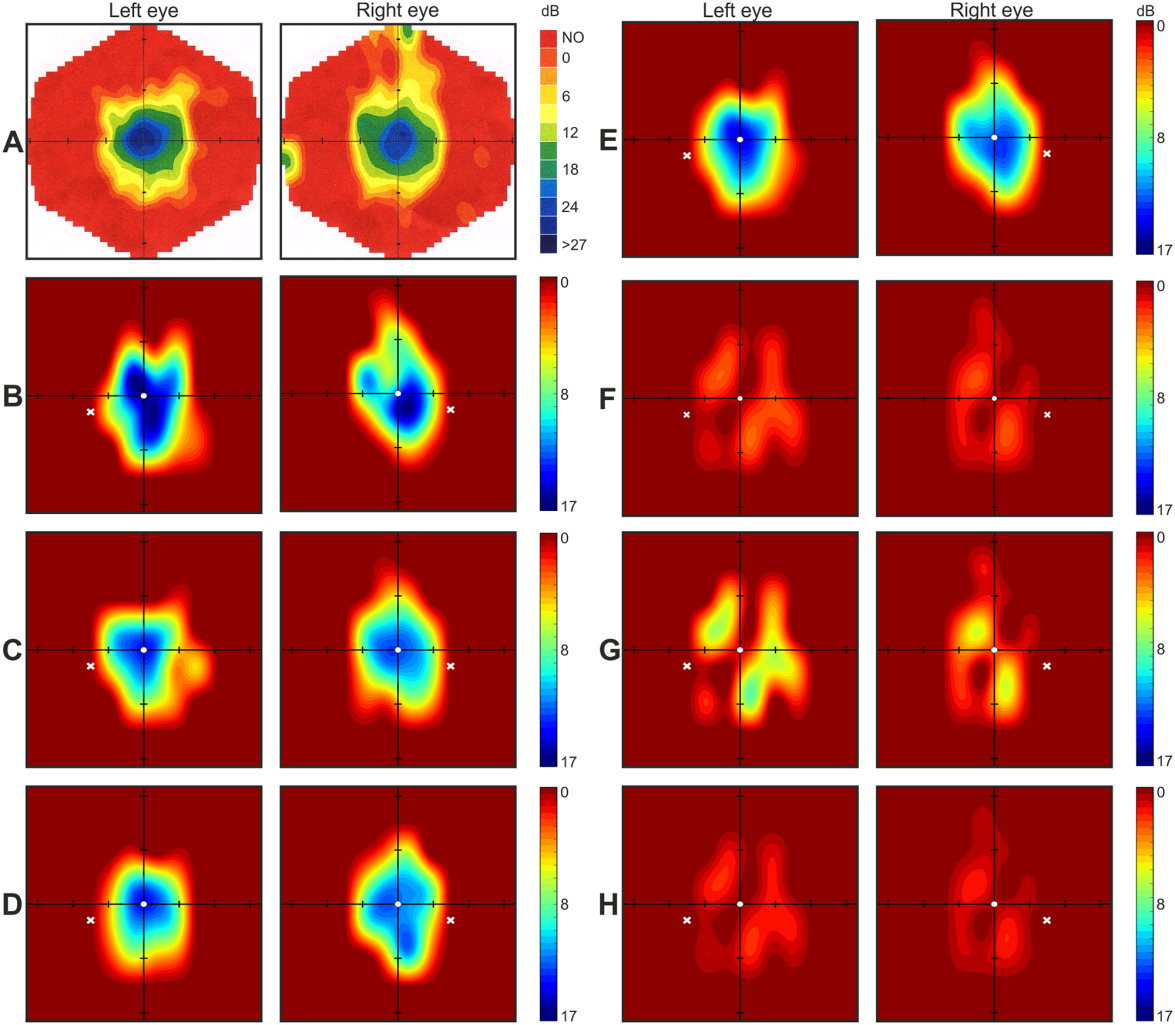
Retinitis pigmentosa patient; MM700 and Specvis visual field graphical maps. **A**. The results from MM700 were mapped according to a decibel scale where NO indicates a lack of response to the stimulus in predefined locations. **B, C and D**. Visual field graphical maps obtained with the Specvis application in three subsequent tests. Color scale of the Specvis maps is expressed in dB. The white X marker indicates the location of predefined, assumed blind spot location. Axes intersect at the fixation point with tick marks at 10° intervals. **E**. Average over three subsequent Specvis tests. **F, G and H**. Standard deviation (SD), variance (SD^2^) and standard error of the mean (SEM), respectively, for three subsequent tests.

Graphically mapped central visual field tested with Specvis vs. results obtained with MM700 are presented in Fig 3. As in the examples of glaucomatous patients, also in the case of retinitis pigmentosa patient, Specvis allowed easy identification of the areas of zero or very low visual sensitivity to stimuli in the visual field, similar to MM700. Because of advanced progress of the retinitis pigmentosa deficit, it was impossible to map blind spot location with the use of either MM700 or Specvis. However, due to retinitis pigmentosa patient’s tunnel vision it was possible to map her central visual field area. Our results indicate that Specvis performed well in the examination of preserved visual field of the retinitis pigmentosa patient. What is important, both variance (SD^2^) and standard error of the mean (SEM) were relatively low for both eyes: SD^2^ = 0.61 and SEM = 0.20 for the left eye; SD^2^ = 0.36 and SEM = 0.15 for the right eye.

#### Stroke patients

Similarly to the retinitis pigmentosa patient, stroke patients were tested once with MM700 for both eyes, and three times for both eyes with Specvis. Average test duration with MM700 was 6:56.5 ± 40.8 s, whereas for tests conducted with Specvis it was 10:12.1 ± 11.4 s.

The average fixation accuracy was very good in both, MM700 and Specvis, i.e. 89.2% ± 8.9% and 91.8% ± 11.6%. As mentioned before, for stroke patients we have used fixation monitor technique *Both*. The FPRR was lower in MM700 than in Specvis, i.e. 2.0 ± 2.0 and 10.5 ± 9.6. This difference results mainly due to the FPRR of stroke patient no. 2, which several times forget to release response key on the keyboard after responding to the visual stimulus.

**Fig 4:**
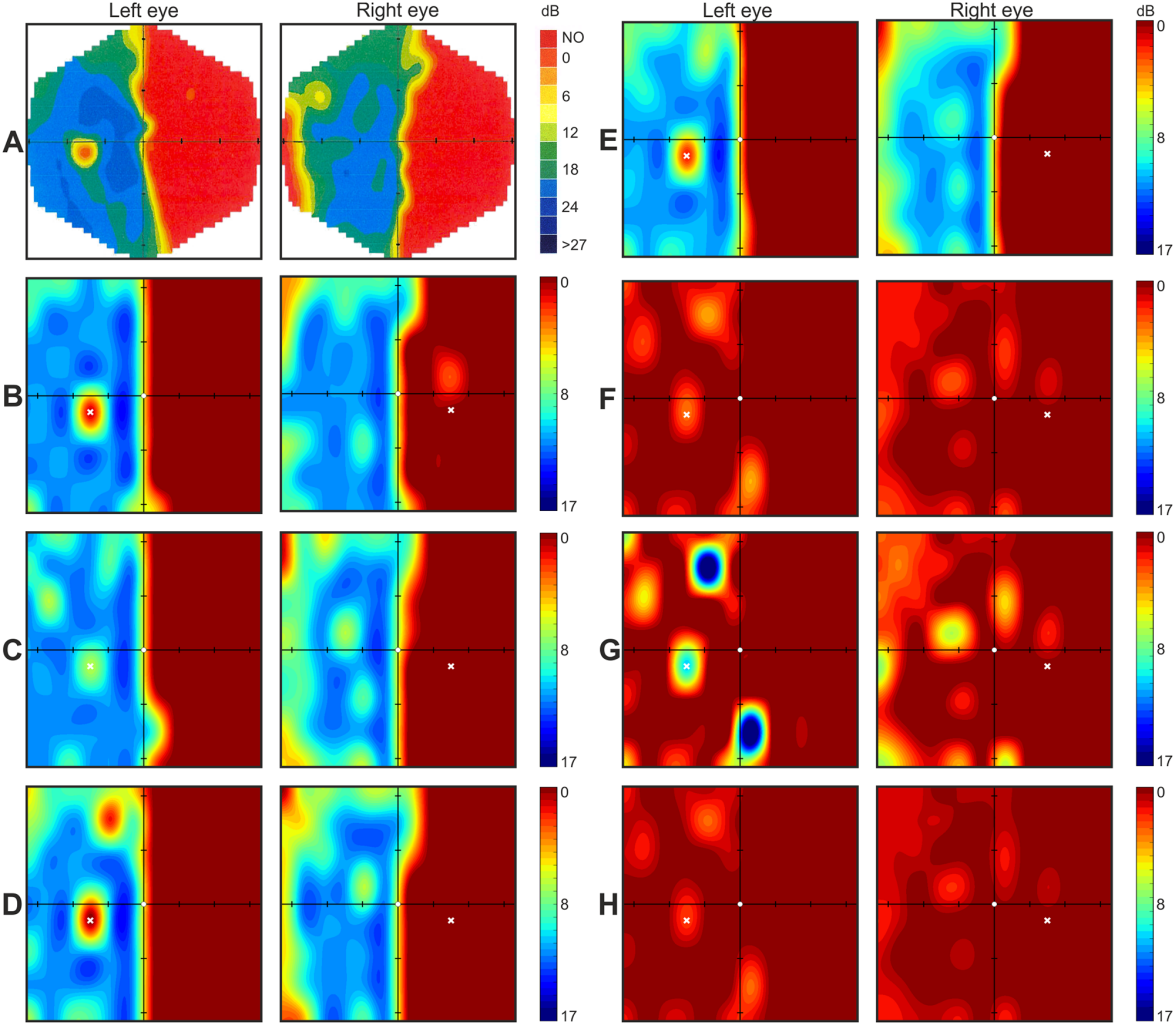
Stroke patient no. 1; MM700 and Specvis visual field graphical maps. The conventions are the same as in Fig 3.

Graphical mapped central visual field tested with Specvis vs. results obtained with MM700 are presented in Fig 4 (stroke patient no. 1) and S4 Fig (stroke patient no. 2). Also here, in stroke patients, Specvis allowed for easy identification of areas of zero or very low sensitivity in the visual field, similar to MM700. The use of both visual field mapping solutions allowed for diagnosis of homonymous right hemianopia resulting from neural deficit caused by ischemic stroke located in the occipital region of the left hemisphere. What is important, in case of both stroke patients, there was no problem with maintaining the fixation throughout the study. In addition, in both patients there is easily distinguishable optic disc location in the left eye. Finally, SD^2^ and SEM for both patients for each eye were as follows: 1) patient no. 1 –SD^2^ = 1.13 and SEM = 0.32 for left eye; SD^2^ = 1.07 and SEM = 0.41 for right eye; 2) patient no. 2 –SD^2^ = 2.08 and SEM = 0.64 for left eye; SD^2^ = 2.06 and SEM = 0.64 for right eye.

## Specvis test-retest reliability

In order to explore Specvis test-retest reliability we have tested 21 healthy subjects (12 females) equally and randomly divided into three groups. The subjects in each group were tested six times for only left eye with the use of different fixation monitor technique, i.e. *Blindspot* (group “B”), *Fixation point change* (group “F”), and *Both* (group “BF”). Subjects had no known ophthalmic, neurological or psychiatric deficits, as well as had normal or corrected to normal visual acuity. Subjects provided written informed consent concerning participation in this study. The study adhered to the Declaration of Helsinki and was approved by the Ethical Committee of the Institute of Psychology of the Polish Academy of Sciences.

Subjects age and visual acuity information are provided in Table 3. Conductance of six tests of the left eye was split to two subsequent experimental days in order to decrease fatigue of the tested eye (each day three tests were performed). Tests were conducted in a darkened room with an inter-test interval of ~5 minutes. The subject’s right eye was covered by an elastic eye patch. The hardware configuration and Specvis settings were identical to those used for retinitis pigmentosa and stroke patients, with the exception of fixation monitor technique, which differs depending of the group.

**Table 3:** Age and visual acuity information of subjects from all three groups each using different fixation monitor technique, i.e. *Blindspot*, *Fixation point change*, and *Both* (this technique consists of Blindspot and Fixation point change techniques). Visual acuity is provided only for the left eye. Visual acuity was tested at the Nencki Institute of Experimental Biology of the Polish Academy of Sciences in Warsaw with the use of ETDRS charts (5 m for BCVA/UVA and 33 cm for CNVA/UNVA). Visual acuity results are expressed on Visus scale (also known as Snellen scale). Twelve subjects wore glasses for distant vision correction. One subject wore glasses for near vision correction. CNVA was measured for all subjects who wore any type of glasses.

Fixation monitor technique	Subject	Age	BCVA	UVA	CNVA	UNVA
**Blindspot**	1	27	0.79	0.63	0.79	1.00
	2	27	1.00	0.10	1.00	0.32
	3	21	1.20	0.13	1.00	1.00
	4	24	1.20	0.10	1.00	0.63
	5	27	N/A	1.58	1.00	0.79
	6	22	N/A	0.79	N/A	0.63
	7	27	1.00	0.10	1.00	1.00
**Fixation point change**	8	29	N/A	1.20	N/A	0.79
	9	25	N/A	1.00	N/A	1.00
	10	24	0.63	0.10	1.00	0.20
	11	21	N/A	1.00	N/A	0.79
	12	21	N/A	1.00	N/A	0.63
	13	29	1.00	0.16	0.79	1.00
	14	30	N/A	1.00	N/A	0.63
**Both**	15	26	1.00	0.10	1.00	0.13
	16	28	1.20	0.32	1.00	0.40
	17	28	1.00	0.63	1.00	1.00
	18	23	0.50	0.10	0.79	0.63
	19	26	N/A	0.63	N/A	1.00
	20	23	N/A	1.20	N/A	1.00
	21	30	1.20	0.20	1.00	0.32
**Average**		25.6	0.977	0.575	0.952	0.709
**SD**		3.0	0.231	0.474	0.092	0.293

Abbreviations BCVA, UVA, CNVA and UNVA means the same as in the Table 1.

### Results

For all subjects for all individual tests from each group we have measured fixation accuracy, FPRR and test duration. Averaged data are shown in Table 4. Detailed data for each individual test for each individual subject are included in S3 Table. Averaged fixation accuracy for each group, i.e. “B”, “F” and “BF”, was 96.89% ± 1.72%, 99.01% ± 0.99%, 88.97% ± 9.59% respectively. Averaged FPRR for group “B”, “F” and “BF” was 0.31% ± 0.42%, 2.26% ± 4.64%, and 1.84% ± 3.11%, respectively, and averaged test duration was 10:11.1 ± 11.1 s, 10:33.9 ± 45.7 s, and 11:14.4 ± 12.1 s, respectively.

**Table 4:** Summary averaged test duration, fixation accuracy (FA) and false-positive response rate (FPRR) for individual subjects from all three groups each using different fixation monitor technique, i.e. *Blindspot*, *Fixation point change*, and *Both*. Averaging was performed for the results from six individual inter-subject visual field examinations.

Subject	*Blindspot*			Subject	*Fixation point change*			Subject	*Both*			
	Duration	FA	FPRR		Duration	FA	FPRR		Duration	FA*	FA**	FPRR
1	10:08.3	48.0/49.3 (97.3)	0.3/283.0 (0.1)	8	10:20.8	43.8/44.3 (98.9)	2.5/324.7 (0.8)	15	11:31.8	11.3/24.5 (46.3)	23.7/23.8 (99.3)	1.5/308.8 (0.5)
2	09:53.2	45.5/46.8 (97.2)	0.2/252.0 (0.1)	9	10:12.2	49.2/49.3 (99.7)	0.5/324.7 (0.2)	16	11:13.0	23.2/24.0 (97.2)	23.0/23.0 (100.0)	0.8/305.3 (0.3)
3	10:14.3	48.2/49.2 (98.0)	0.5/266.5 (0.2)	10	10:11.8	45.7/47.0 (97.2)	62.8/320.3 (13.6)	17	11:04.2	21.8/25.2 (87.2)	23.0/23.2 (99.3)	1.2/308.8 (0.4)
4	10:24.7	48.2/50.5 (95.4)	3.7/267.7 (1.3)	11	10:29.3	48.7/49.7 (98.0)	2.0/322.7 (0.6)	18	11:28.3	20.0/24.7 (81.0)	23.5/23.8 (98.7)	43.8/270.3 (9.4)
5	10:05.3	46.2/47.7 (96.9)	0.2/274.0 (0.1)	12	10:15.2	48.3/48.7 (99.3)	0.3/325.2 (0.1)	19	11:14.5	13.3/23.8 (56.1)	22.8/23.3 (97.9)	4.5/304.5 (1.4)
6	10:24.8	45.7/48.7 (93.8)	1.2/279.0 (0.4)	13	10:11.2	49.3/49.3 (100.0)	0.2/304.8 (0.1)	20	10:58.3	23.5/24.2 (97.2)	22.7/23.0 (98.6)	0.5/303.0 (0.2)
7	10:07.3	48.8/49.0 (99.6)	0.0/261.3 (0.0)	14	12:16.5	49.0/49.0 (100.0)	1.2/319.7 (0.4)	21	11:10.3	22.2/25.5 (86.9)	24.3/24.3 (100.0)	2.2/291.0 (0.7)
**Average**	10:11.1	47.23/48.74 (96.89)	0.87/269.07 (0.31)		10:33.9	47.71/48.19 (99.01)	9.83/320.30 (2.26)		11:14.3	19.33/24.56 (78.84)	23.29/23.49 (99.11)	7.79/298.81 (1.84)
**SD**	00:11.2	1.27/1.10 (1.72)	1.21/9.84 (0.42)		00:45.7	1.97/1.78 (0.99)	21.60/6.65 (4.64)		00:12.1	4.60/0.58 (18.48)	0.53/0.45 (0.71)	14.75/12.90 (3.11)

The expression of FA and FPRR values is the same as described in the legend of Table 2;

*—FA measured by technique *Blindspot*;

**—FA measured by technique *Fixation point change*.

In this place it is important to emphasize, that *Blindspot* (used in group “B” and partially in group “BF”) fixation monitor technique use display of *control* stimulus in predefined location, which we assume should correspond to optic disc’s representation. For all subjects from all groups this location was the same, i.e. 15° temporally and 3° ventrally from the fixation point. However, location of optic discs representation varied between subjects. Manual mapping of blind spot revealed, that for some subjects (e.g. subject no. 19) *control* stimulus was displayed outside assumed optic disc representation in the visual field. This caused very low fixation accuracy measured with the use of *Blindspot* technique. Thus it is important to assess individual blind spot representation in the visual field, when using *Blindspot* or *Both* fixation monitor techniques, if we want visual field examination results to be considered as reliable, even when obtained results in a form of visual field graphical map looks “reliable”.

**Fig 5:**
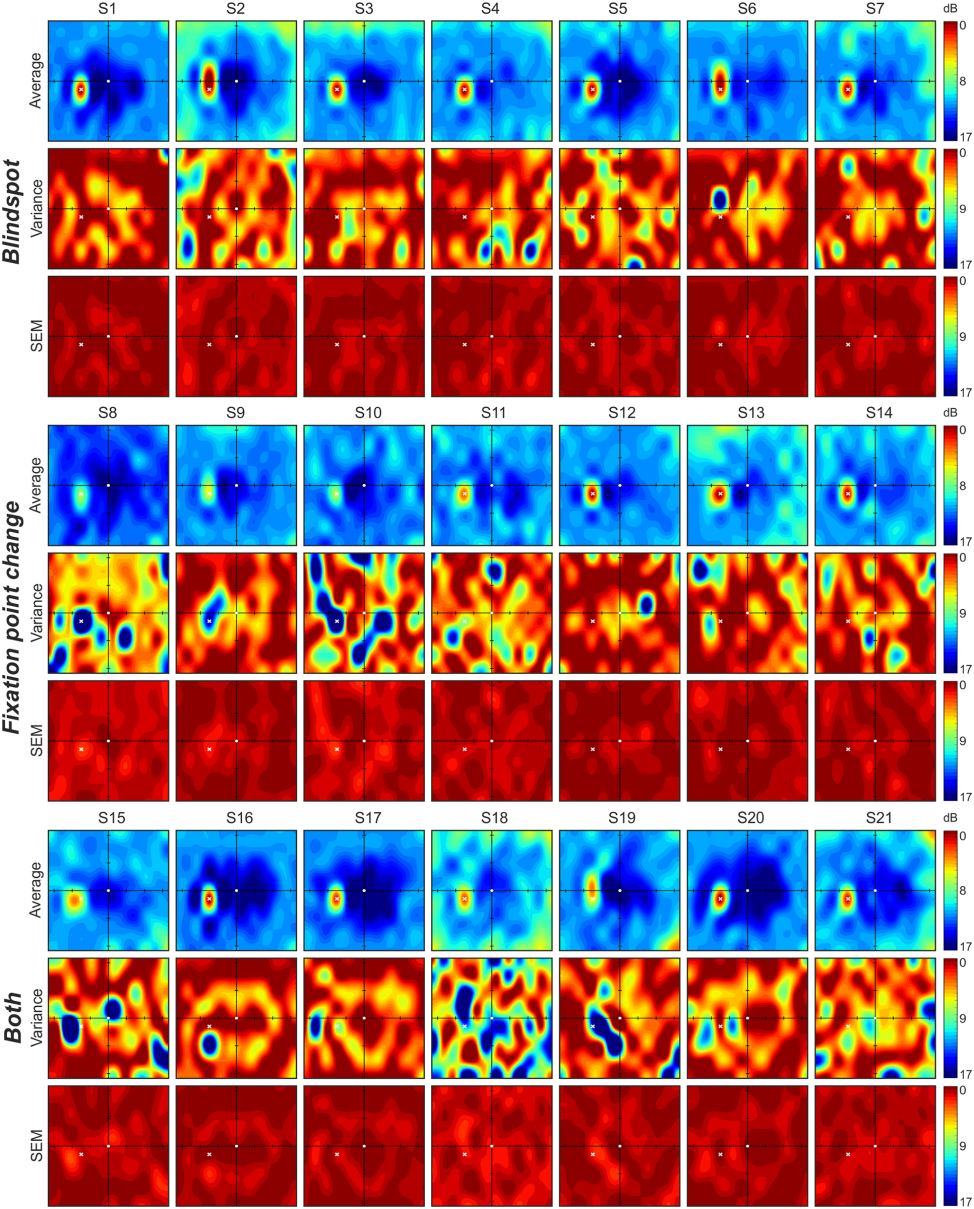
Summary results for all 21 subjects (S1-S21) from all three groups each using different fixation monitor technique, i.e. *Blindspot*, *Fixation point change*, and *Both* (this technique consists of *Blindspot* and *Fixation point change* techniques). Average, variance and SEM visual field maps calculated for six individual Specvis tests for all subjects from all groups. Conventions are the same, as in the Fig 3.

**Fig 6:**
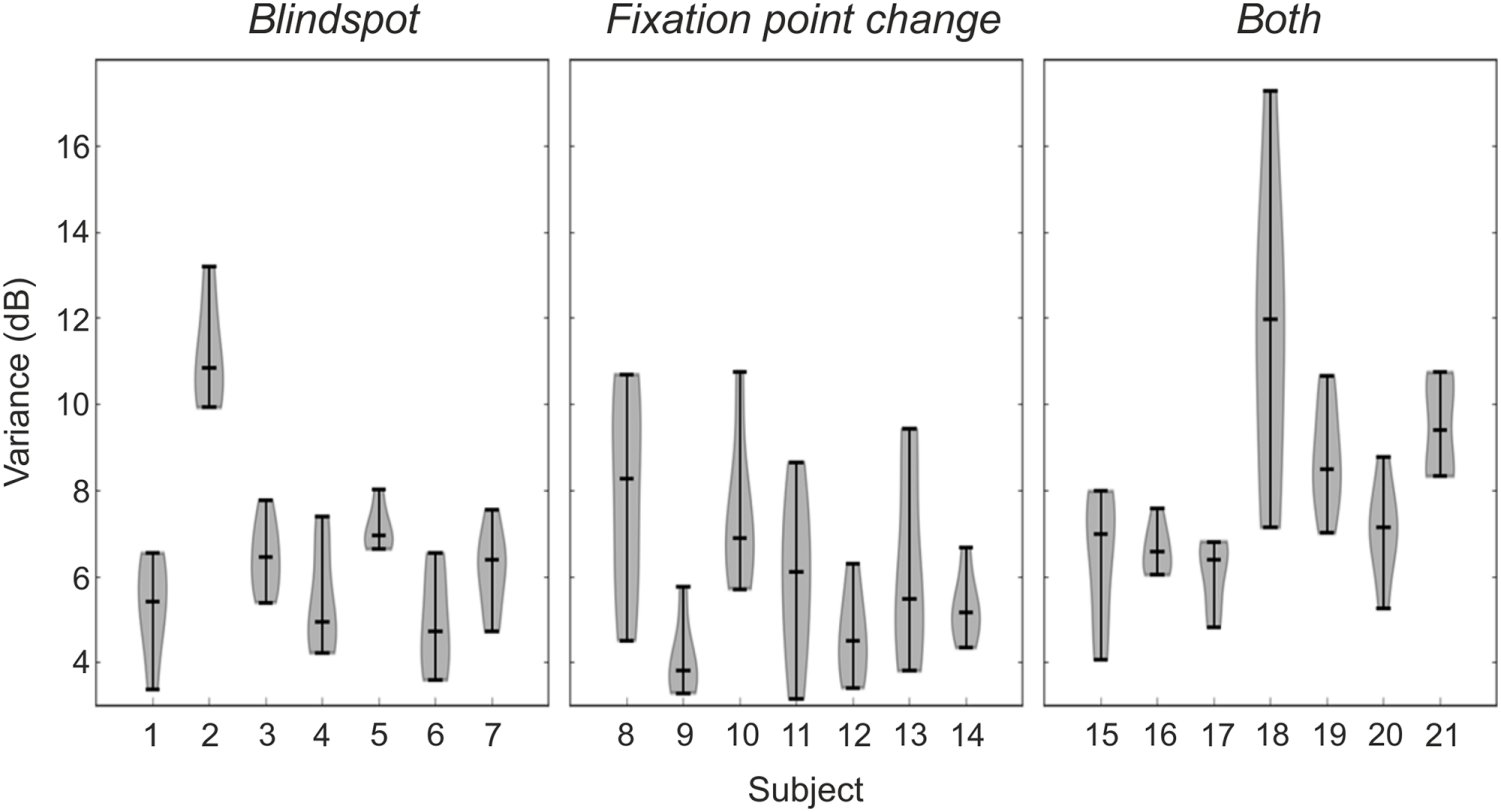
Intra-test variance for all visual field tests, subjects, and groups each using different fixation monitor technique, i.e. *Blindspot*, *Fixation point change*, and *Both*. Each subject’s violin consists of six global variance values calculated across all six visual field graphical maps. Middle line present in each violin plot represents the median. Bottom and top “whiskers” of the plots are variance extrema. The smaller the spread between the extrema, the lower inter-test variance.

Going further, we have also calculated average, variance, and standard error of the mean, across visual field graphical maps from six individual tests for each subject and present the results in Figs 5 and 6. As mentioned before in the context of patients with ophthalmic deficits, Specvis has no problem with finding areas of zero or low visual sensitivity to visual stimuli, which resulted in accurate mapping of the blind spot in the visual field almost in all subjects. However, variance between individual tests occurs, and in the case of subjects 8, 10, 11, 13 and 18 it was fairly high, what can be partly explained by low fixation accuracy and high FPRR, as well as individual differences in capabilities for maintaining the fixation during whole test.

**Fig 7:**
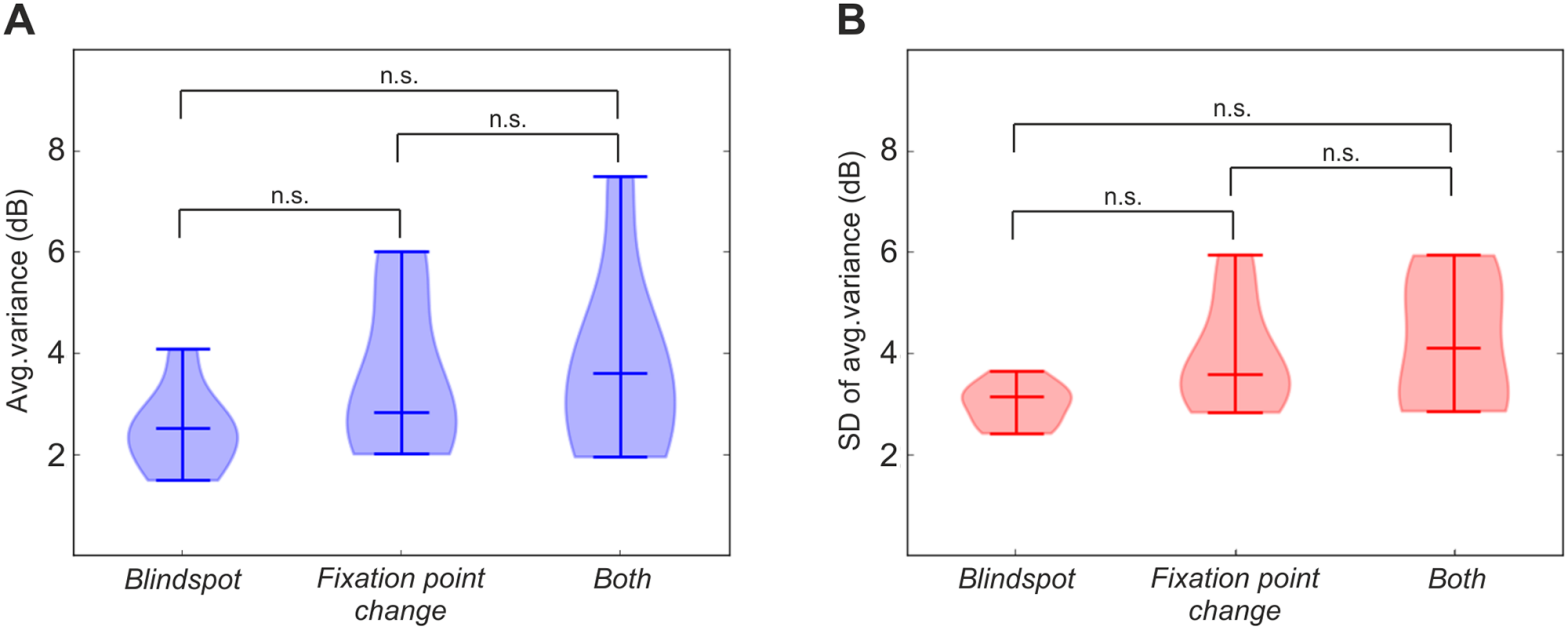
Violin plots for average variances (A) and standard deviation of average variances (B) calculated across groups each using different fixation monitor technique, i.e. *Blindspot*, *Fixation point change*, and *Both*. Values are expressed in decibels (dB). Conventions are the same, as in the Fig 6. T-test for the means of two independent samples with Welch’s correction for inequality of variances was calculated for paired groups. Results were insignificant (n.s.) with the assumed significance threshold p < 0.05 even without Bonferroni correction for multiple comparisons.

We have also compared average variances and their standard deviations between groups, in order to check whether fixation monitor technique changes significantly test reliability (Fig 7). We do not have found any significant differences between fixation monitor techniques in the context of inter-test reliability. However, taking into account individual differences in blind spot representation location in visual field, we suggest to use fixation monitor technique *Both*, which incorporates displaying *control* stimulus in predefined blind spot location, as well as changes of the fixation point characteristics.

## Comparing Specvis results from two subsequent visual field tests

Specvis allows to compare the visual field examination results from two subsequent tests. This functionality can be useful especially in the context of following the improvement or deterioration of the patient’s vision in various visual impairments, such as glaucoma, retinitis pigmentosa or stroke. At this point of Specvis development (version 1.1.0) it is possible to compare the results from two subsequent tests performed with the use of the same settings by means of simple subtraction. This “comparing” functionality is available from the level of Specvis graphical user interface in patient’s results preview window (S10 Fig) (for more details see section ‘Software implementation‘).

**Fig 8:**
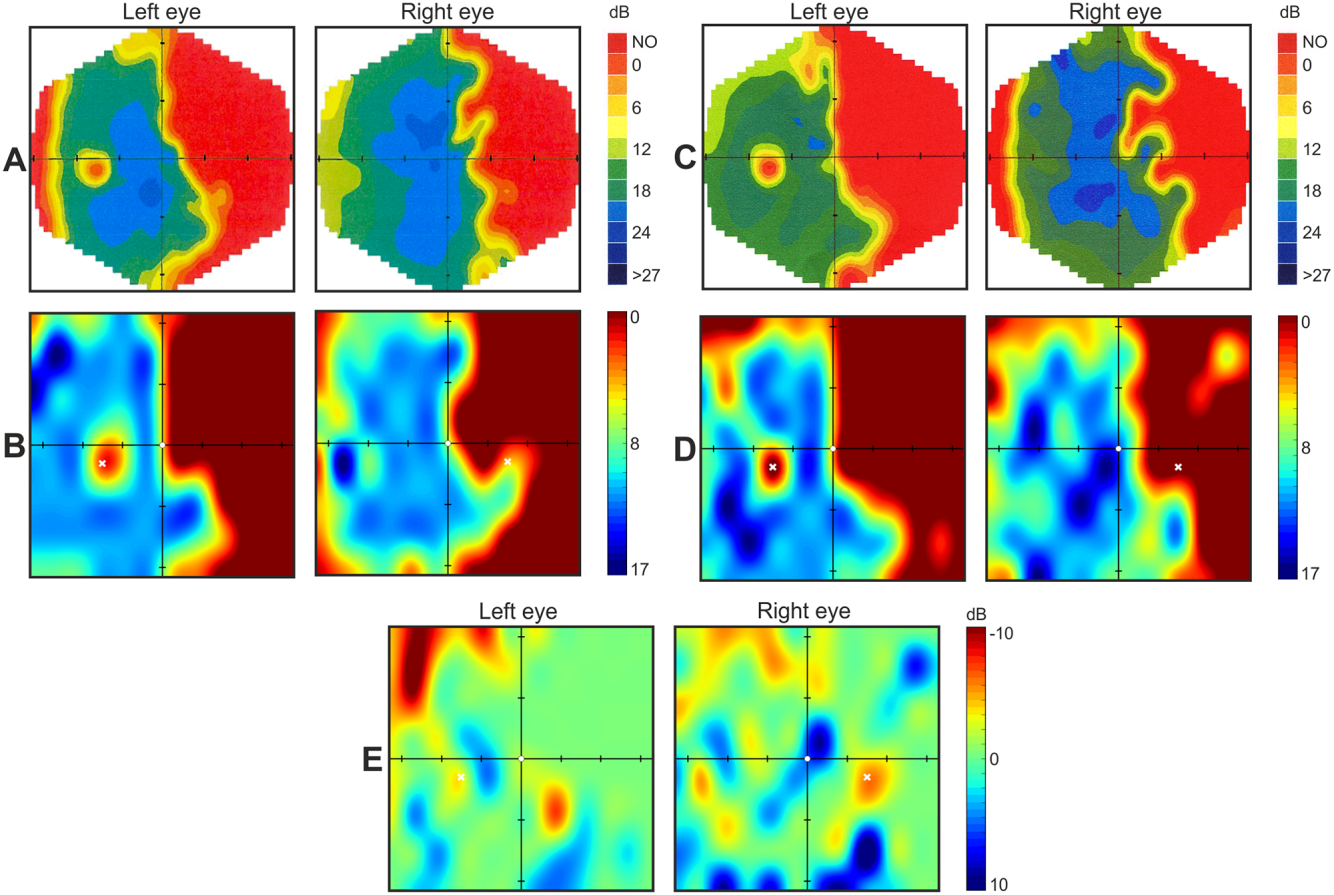
Comparison of Specvis results from two separate visual field examination days. **A and B**. MM700 and Specvis results (respectively) acquired in one day, say “day zero”. **C and D**. MM700 and Specvis results (respectively) acquired also in one day, but 20 days after “day zero”. **E**. Comparison of Specvis results from both aforementioned days by means of subtraction. Conventions are the same as in the Fig 3.

In order to present this functionality in action, we compared the results of stroke patient no. 2 obtained in two consecutive tests spread by 20 days (Fig 8). The patient was tested with MM700 and Specvis for both eyes. The Specvis results from the first test were subtracted from the Specvis results from the second test for each eye separately. Simple eye-based evaluation of the comparison can suggest subtle improvement of vision in bottom part of the lower-right quadrant in both eyes, as well as delicate deterioration of vision in upper part of the upper-left quadrant, also in both eyes. However, taken into account test-retest variance in both, MM700 and Specvis, we should compare averages consisted of the results from few subsequent tests, rather than compare the results from two single tests. Nevertheless, even such simple functionality for performing comparison between the results from two subsequent tests can be useful in fast and easy assessment of the improvement/deterioration of the patient’s vision.

## Discussion

Our main goal was to create a supplementary method for visual field examination that could fill the gap between confrontation and perimetry tests. We wanted this method to be widely available, affordable, easy to use, and importantly, reliable. In order to meet these requirements, we have written the Specvis application in the Java programming language and made it free and open-source. Specvis can be run on any personal computer with any operating system, and can be managed by anyone, physician, scientist, or an ordinary unspecified user.

In order to initially verify the reliability and usefulness of the Specvis application to examine visual field, we conducted two short validation studies. We compared visual field sensitivity results of glaucomatous patients when tested with the MM700 and Specvis performed very satisfactorily. The average Specvis test duration was only 34 s longer vs. MM700, but if the Specvis brightness vector length for the procedure *Basic* is reduced from 17 to 13, test duration is reduced by 4.5 minutes. Results from the glaucomatous patient no. 4, for which brightness vector length equal to 13, clearly indicate the presence of the same visual field deficits as seen with the MM700. Thus, it is worth studying further how the length of the brightness vector in the *Basic* procedure impacts on the accuracy of the evaluation of visual field condition, so it would be possible to reduce test duration even more. In our opinion, a brightness vector length equal to 13, or even 9, is sufficient to conduct fast screening tests aiming to assess the general condition of the patient’s visual field, while emphasizing that the search for areas of zero sensitivity in both Specvis and MM700, can be seen easily.

The usefulness of the shortest brightness vector length equal to 9 can be extrapolated from the results of the retinitis pigmentosa and stroke patients, where this sensitivity level of the test procedure was sufficient for detecting visual field areas of zero- and low-sensitivity to visual stimuli. What is important, the results obtained with the use of Specvis application do not differ from those obtained with the MM700. This is not only in the case of graphical maps, but also fixation accuracy, FPRR as well as test duration. Of course there are some subtle differences. For example, in stroke patients test duration was shorter by ~3 minutes in favor of MM700.

We have also evaluated the test-retest reliability of Specvis based on 21 healthy subjects. The results suggest that Specvis has a low test-retest variability. However, inter-test intrapersonal variability can differ from person to person. Thus, it is important not only to ensure subject’s concentration during the study, but also to develop solutions and functionalities which will help to decrease and stabilize inter-test variability across all subjects, independently of health condition and environmental variables.

In summary, Specvis can be used as a reliable visual field examination tool, especially in the search for areas of zero- and low-sensitivity to visual stimuli. The test duration depends not only on the application settings, but also on patient capabilities. Nevertheless, the total test duration with Specvis is not significantly different from that of MM700 and as we have shown in the case of retinitis pigmentosa patient it can be even shorter. The fast screening test for general condition of the visual field seems to be the best way to use Specvis. This fits perfectly our initial requirement that such a program should fill the gap between confrontation and perimetry tests. We believe that Specvis can be used for clinical/diagnostic purposes (especially in underdeveloped countries), but also in the scientific research that need to control for visual field losses.

Although the *Basic* procedure is well suited to the visual field examination types covered in this paper, we are aware that Specvis needs further development and improvement. For example, currently a photometer is required to configure new luminance scales thus placing an extra burden on the end user. It must be noted that it is required only when one needs an exact information about the range of tested luminance values expressed, e.g., in cd/m^2^. Furthermore, the *Basic* procedure is a staircase procedure, thus sensitive to the situation, when the subject (i) responded to the stimulus which should not be perceived, or (ii) did not respond to the stimulus which should be perceived. The number of these two types of errors should be limited when the subject maintains their fixation, but anyway, the procedure *Basic* is unaware of those errors and “treats” them as a valid responses to the stimuli presentation. Thus, it is the examiner responsibility to assess the reliability of the visual field results based on fixation accuracy rate and FPRR, and repeat the test if necessary. Therefore, our aim is constant development, improvement and maintenance of the Specvis application that will deal with the situations described above, as well as developing new, more reliable testing procedures and algorithms. Additional feedback from interested users will in the future help to solve problems with the application and provide ideas that will improve the software and end-user experience.

Results of subject examination with the current version of Specvis using simple detection task provide predominantly information about luminance threshold for local detection of visual stimuli, thus allow for assessment of the visual field extension. However the spatial changes in the visual field are not the sole deficits in visual impairment. For example, in glaucoma, the changes in the size of visual field may be accompanied by sleep disorders, deficits in visuo-motor coordination and in temporal processing of visual information (for review see [26]). Growing body of research indicates that temporal deficits in vision may constitute important aspect of perceptual impairment.

Studies of response properties of neurons at different stages of the visual system obtained in animals following structural damaged to the visual system either by performing retinal lesion or applying a pressure to optic nerve indicate deficits in perception of fast visual stimuli [28–31]. In animal research, the deterioration in temporal processing has been shown in middle temporal cortex of aged rhesus monkeys [32]. Age related changes of temporal frequency tuning suggested slower with age temporal processing of visual information. In human studies slower reaction time has been shown in patients with visual field loss due to pre- or post-chiasmatic damage of the visual pathway [33]. Deterioration of temporal processing of visual information has been found also in patients with optic neuritis not showing visual field loss [34–36]. Taking this into account, the future development of the Specvis will aim to include the possibility of assessment the temporal aspect of visual processing, such as temporal resolution, cut-off temporal frequency and reaction time.

We are also aware of problems with visual field examination in patients who have central visual field deficits, or who cannot maintain properly the central fixation point. One of the example are people with Leber’s hereditary optic neuropathy which affects the central visual field in one or both eyes [37]. The problem with visual field examination of people with impaired central vision is not trivial and can be approached from many directions. One of them can be based on not expensive method of monitoring position of the tested eye and providing simultaneous acoustic cues to the subject. This eye-tracking-based solution can be also augmented by adjusting locations of visual stimuli on the screen—based on the eye-tracker readings, positions of all elements displayed on the screen are adjusted according to the gaze of the examined eye. Both solutions can be implemented in Specvis application. Further, our aim in the nearest future is to transfer the Specvis from personal computers to virtual reality, which is much more vital environment for visual system diagnostics.

## Software implementation

### Source code, requirements and availability

Specvis was written in the Java programming language [38, 39] and it’s version 1.1 requires installation of the Java Runtime Environment (JRE) in version 8u121 or above. The latest version of JRE can be downloaded from the Oracle’s website (http://www.oracle.com/technetwork/java/javase/downloads/jre8-downloads-2133155.html).

After JRE installation or checking the JRE version by typing in the terminal or command window ‘java -version’, the current version of Specvis can be downloaded from www.specvis.pl. The website is linked with a GitHub repository where the complete Specvis source code is located (https://github.com/piotrdzwiniel/Specvis). The repository will always contain the latest version of Specvis. The source code and the application itself are released under the terms of GNU General Public License in version 3 as published by the Free Software Foundation [40]. Software covered by this license is and will be free and open-source. In general, this license ensures, that everyone can use the software and modify it, however, each new release is also covered by the same license, so the freedom and capacity of the software remains preserved.

### Description of the downloaded package and first launch of the application

The Specvis website downloadable *.zip file contains three files and two directories. These are: *Specvis*.*jar*, *patients*.*s*, and *screenLuminanceScales*.*s*. The directories are: *Results* and *Settings*.

*Specvis*.*jar* is an executable *.jar file used to run Specvis application, and is a package aggregating the compiled application’s source code and all related content, such as external libraries, images, and data. Depending on the operating system and set options running the application can be done in two ways: 1) simply double click on the *Specvis*.*jar* file; 2) type ‘java—jar Specvis.jar’ in the terminal or command window while in the file location.

The second file *patient*.*s* serves as a type of text database and contains information about the patients added to Specvis. Each row of this file is dedicated to a single patient and can hold the following information: unique ID number (P_yyyyMMdd_xxxx; P stands for patient and xxxx is a string of four random lower-/uppercase letters and numbers), personal information, and any additional information.

The *screenLuminanceScales*.*s* file is similar in structure to the above *patients*.*s* file and serves as a text database. Each row is dedicated to a single scale. In addition to ID information and the scale name, the file contains data from six brightness luminance measurements and any additional information about the scale. Similar to adding a new patient, a new scale creation generates an individual unique ID number for the scale (S_yyyyMMdd_xxxx; where S stands for scale).

The *Results* and *Settings* directories serve as a storage for patient visual field examination results and Specvis settings respectively. It is important not to move, modify or delete any of the aforementioned *.zip archive files or directories. The whole package can be moved to a new location but the relative positions of each file must remain the same together in one folder. All Specvis content and its dependencies are embodied into one single executable *.jar file which is not installable, and can be run directly from external media such as pendrive.

### Patient management and loading the settings

**Fig 9:**
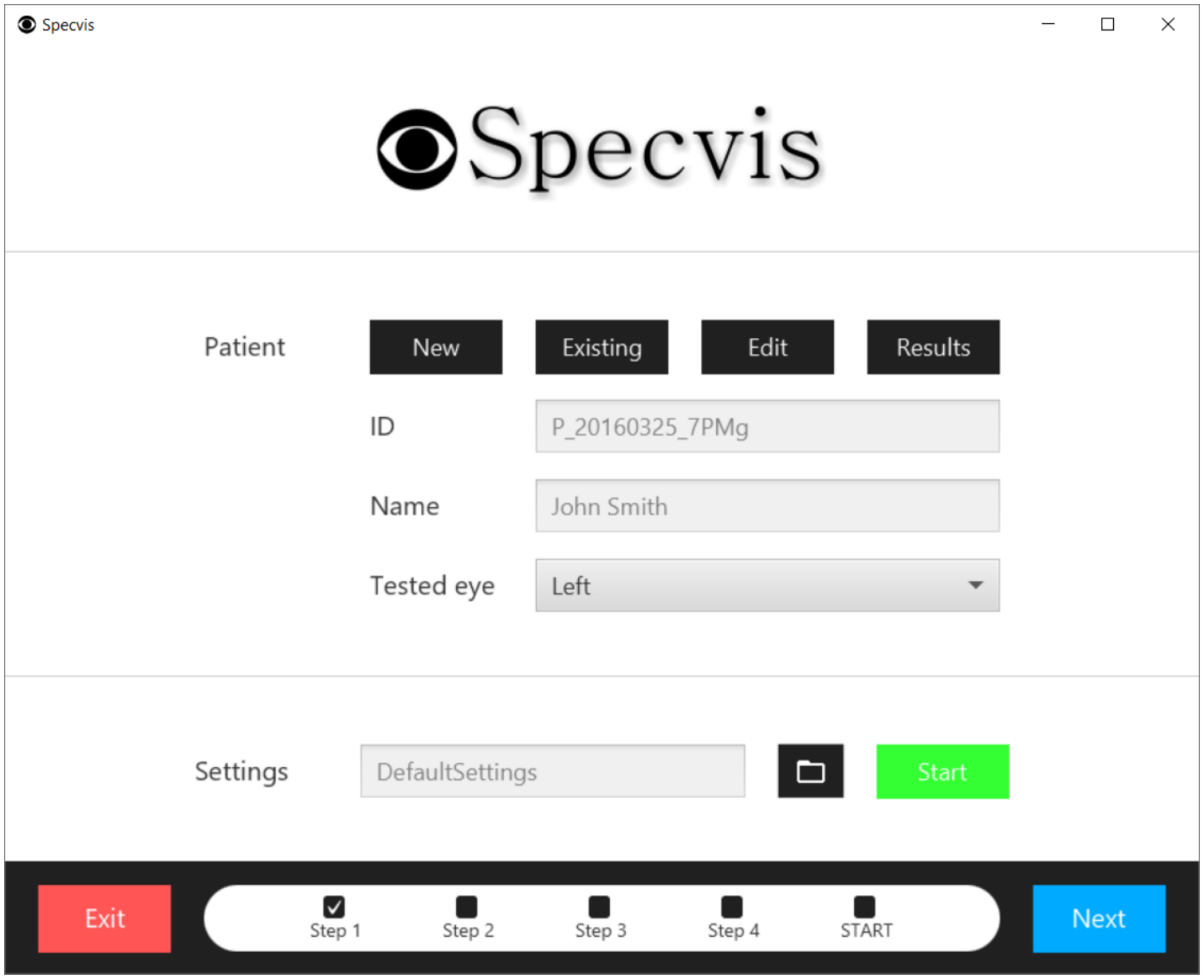
The initial Specvis window. After launching Specvis, this first window will be displayed and the user can add, choose or edit patient details, as well as preview their previous results. It is also possible to load a default template for settings if the user does not wish to do this manually.

**Fig 10:**
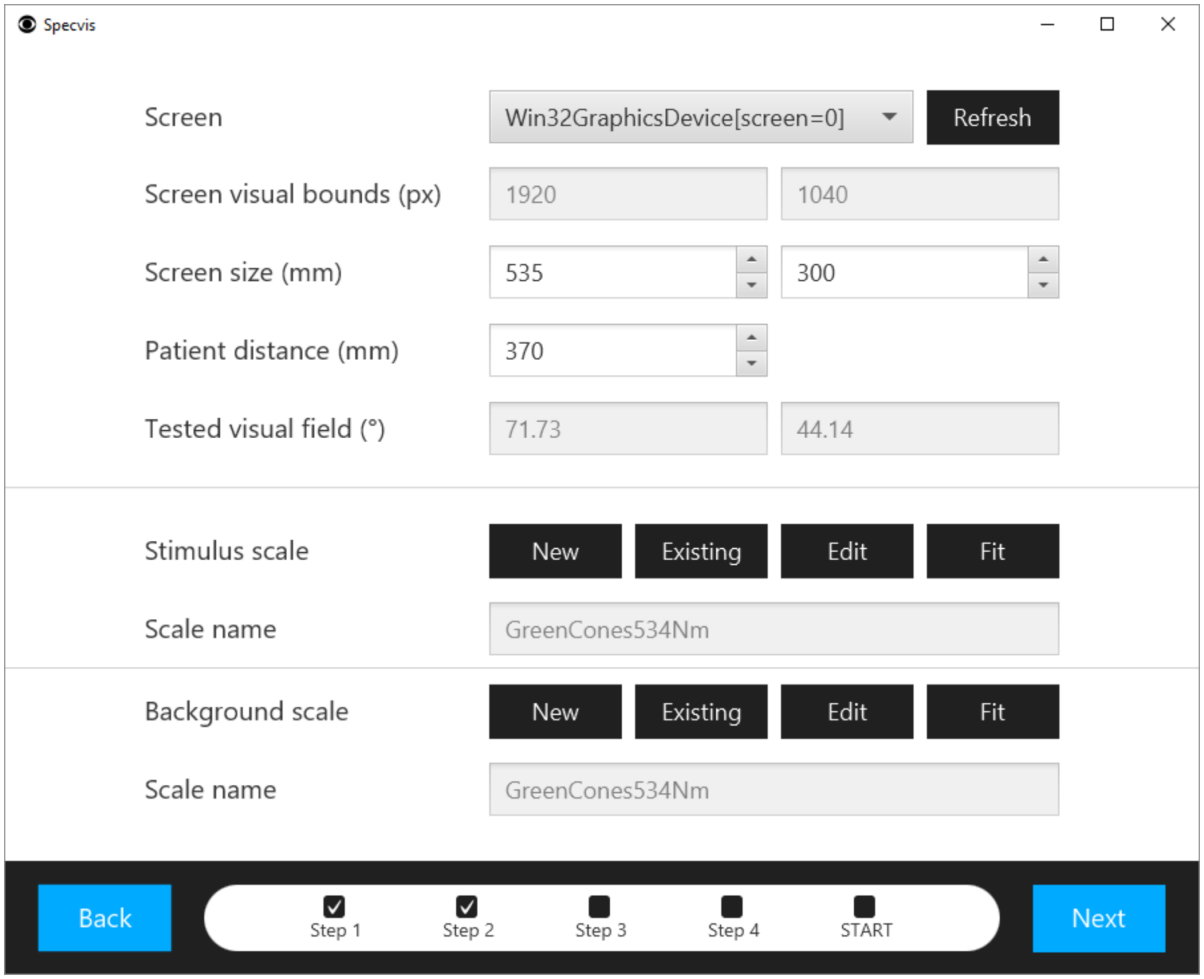
Window for optional adjustment of screen and luminance scales. The user can adjust settings for the chosen screen as well as configure and set luminance scales for the stimulus or background.

**Fig 11:**
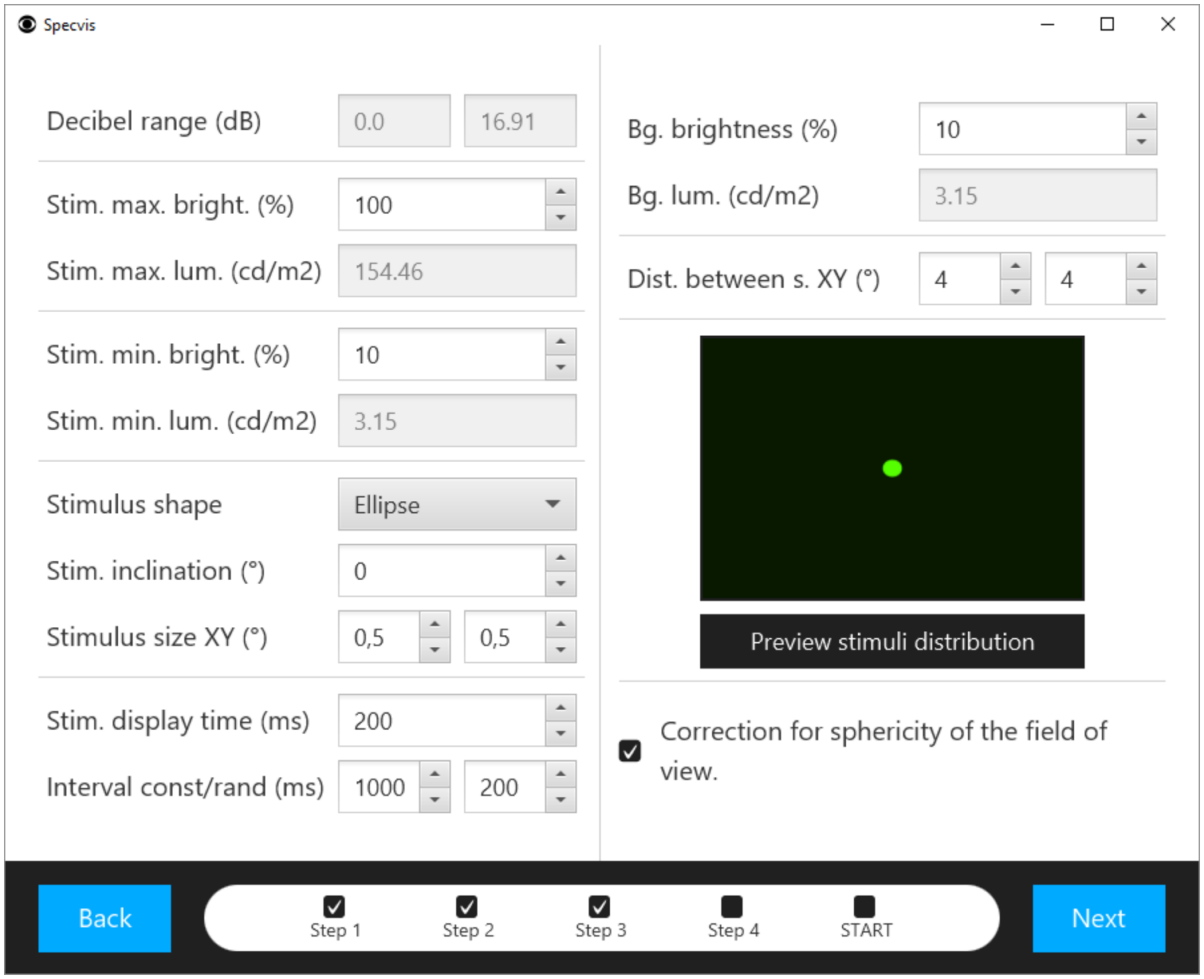
Window used for optional adjustments of the stimulus and background. The user can adjust settings for the stimulus and background, as well as preview the stimulus distribution on the previously chosen screen.

**Fig 12:**
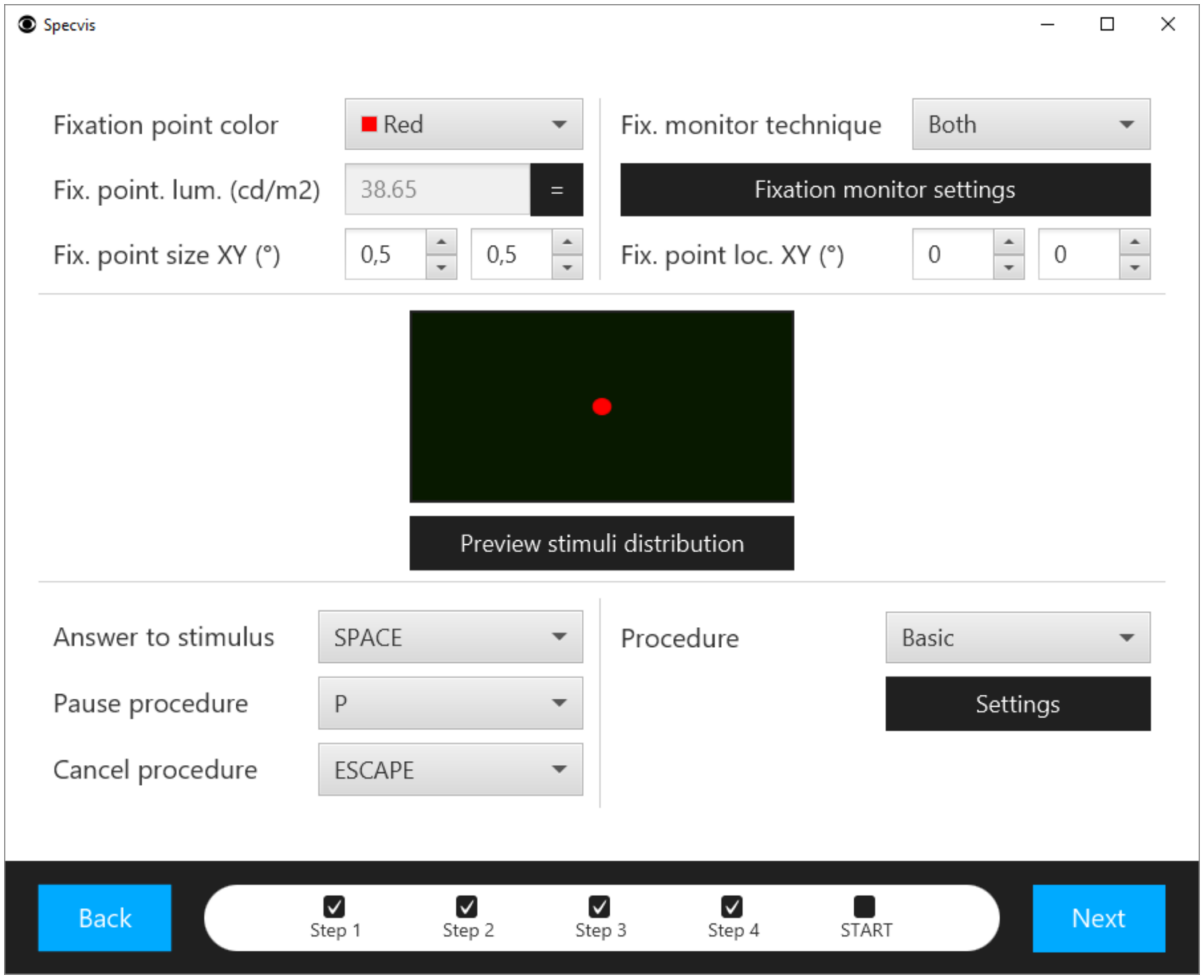
Window for adjusting fixation and other options. In this window the user sets the fixation point characteristics, measure its luminance, and set its position on the screen. At this level, the user can also choose and adjust the fixation monitor technique (*Blindspot*, *Fixation point change*, *Both*), as well as the procedural algorithm (*Basic*) that will be used in the visual field examination test. Keyboard configuration can also be changed here.

After launching Specvis it is possible within the very first window (Fig 9), to choose the patient, edit their personal information, and preview (as well as export or compare) their results from previous visual field examinations or enter new data. It is necessary to select the patient’s eye to be tested and, optionally, choose any existing settings template previously saved in *.sset file format (to save settings see below). Loading the settings will set all the parameters in all Specvis windows where options for the procedure can be set (Figs 10–12 and S7–S9 Figs).

### Screen settings and configuration of the luminance scales

After selection of patient’s eye for examination, it is possible to go to the next Specvis scene (Fig 10), where screen and luminance scales settings adjustments are required. Application provides several predefined scales which can be used in tests by default. Nevertheless, Specvis should be configured before use with new hardware setup and different light conditions.

Specvis uses patient’s visual field degrees of arc to express values of the distance, inclination and size. In order to display elements on the chosen screen appropriately, the program has to calculate two things: 1) the range of the patient’s visual field occupied by the screen and 2) the number of screen pixels/ 1° of arc. This is done by providing information about the screen width and height, and the distance between the patient and screen (all values are expressed in mm). In an ideal situation, the distance from the screen reflects the distance measured from the cornea to the center of the screen. Specvis calculates the extension of visual field occupied by the screen in the horizontal and vertical planes separately as follows:

$$2\theta =2\;\times \text{arctan}\left(\frac{y}{2x}\right)\;\times \;\frac{180}{\pi }$$

where *x* = patient distance from the screen, *y* = screen width or height (depending on whether the horizontal or vertical calculation is being made), and 2*θ* = the extension of patient’s visual field occupied by the screen expressed in degrees of arc. Based on the screen resolution, Specvis then calculates the number screen pixels/ 1° of arc horizontally and vertically (i.e. screen resolution/ 2*θ*).

Screen models differ from each other with respect to hardware, thus individual technical specifications result in different minimum and maximum possible luminance levels for the display (e.g. for one screen the max luminance will be 150 cd/m^2^ where for the other it will be 200 cd/m^2^). Additionally, each model has its own software options for setting the minimum and maximum luminance in part due to adjustments to brightness, contrast and gamma, or other values, thus direct access to the information about luminance values of the screen are not possible without photometer measurements.

Specvis estimates the minimum luminance required for the detection of a specific stimulus throughout the examined visual field. In contrast to Specvis, perimeters are an integrated hardware system where stimulus luminance is known. Specvis has no direct access to information about luminance values and therefore, it is necessary to configure the application for the screen and light conditions being used. The configuration can be done via the Specvis window dedicated to the creation of a new luminance scale (S5 Fig). This is performed in two steps. Firstly, the luminance in cd/m^2^ is measured with a photometer in a six square patterns with a predefined background color, hue and saturation. Brightness is increased in each square from 0 to 20, 40, 60, 80 and 100% and measured with the photometer. Secondly, Specvis then fits a second degree polynomial to the luminance values resulting in the function:

$$f\left(HSB\;brightness\;\left(\%\right)\right)=fitted\;luminance\;\left(cd/m^{2}\right)$$

which is calculated for all brightness values, i.e. 0–100, resulting in a vector of fitted luminance values of the length equal to 101. The quality of the fit is defined by the chi-squared statistic and SD. It can be assumed that the element of a specific color expressed on the hue-saturation-brightness (HSB) space has a certain luminance value, this makes it possible to examine visual sensitivity to stimuli in the visual field on the luminance scale regardless of the chosen hardware, and make the results comparable between different hardware configurations. It is possible to use Specvis without photometer, but it is than impossible to interpret visual field results in the luminance domain.

**Fig 13:**
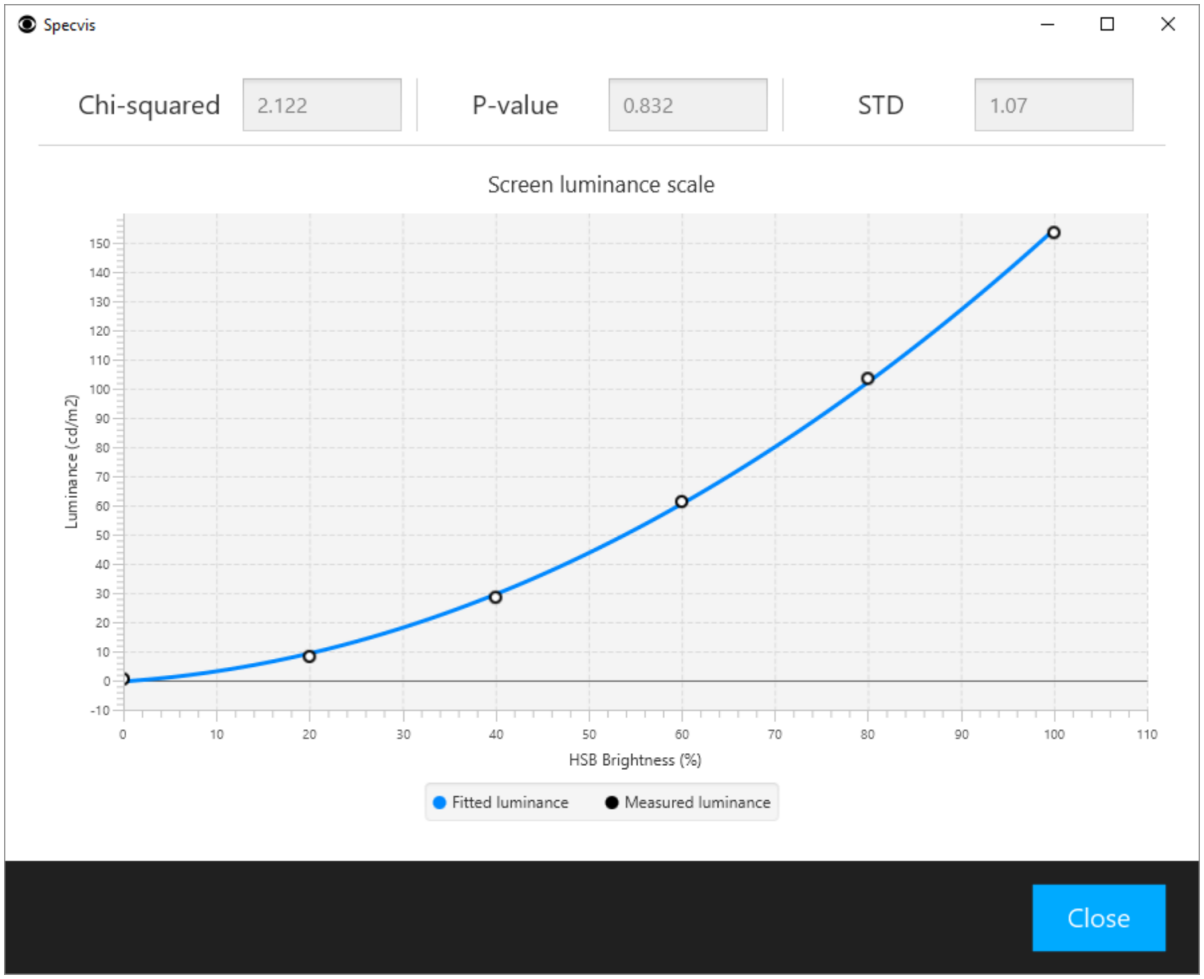
Window for previewing the quality of fit for the chosen luminance scale. The user can check the chosen luminance scale for goodness of fit, in the context of how accurate is the relation between luminance expressed in cd/m^2^ and brightness expressed in the HSB space. The quality of the scale can be assessed based on the chi-squared statistic and its significance (p-value), as well as the standard deviation (SD).

After providing a name for a newly created scale and choosing its hue and saturation, the six luminance measurements described above are obtained and any additional useful information inputted, e.g. screen model, configuration of settings, type of photometer, and ambient light conditions. It is now possible to check how good scale is by checking its quality of fit (Fig 13); it is also possible to edit the scale later. Note that Specvis uses separate luminance scales for the stimulus and background displays. That is why it is necessary to choose existing scales or to create new scales for the stimulus and background. Only after the creation and/or choosing a scale is it possible to proceed further.

### Stimulus and background options

Specvis allows the user to freely adjust stimulus options and the background (Fig 11). It is possible to choose from two different shapes of the stimulus, i.e. *Ellipse* or *Polygon*. The *Polygon* shape used in conjunction with the *Stimulus inclination (°)* can create a stimulus in form of inclined stripes or a simple square, while the *Ellipse* shape creates a simple dot-like Goldmann stimulus as used in various types of ASP. There is the possibility to change the stimulus size and its display time, as well the inter-stimuli interval, either constant or random. Beside these basic settings, the examiner can also set the luminance range for the stimuli in order to measure visual sensitivity to stimuli brightness levels in the visual field. For example, setting *Stimulus maximum* and *minimum brightness (%)* to 100 and to 11 respectively, Specvis will only look for the luminance threshold within this range in a given location despite the chosen procedure. In addition, stimulus density displayed on the screen can be adjusted using the *Distance between stimuli XY (°)* parameter in order to reduce or increase the accuracy of the visual field examination. It is also possible to set the background brightness for its individual luminance scale.

Specvis calculates the dB range for the minimum and maximum stimulus luminance, in line with the currently utilized perimetric systems that use dB values to express visual sensitivity to visual stimuli levels across visual field. This allows comparison across techniques way as well as inter- and intra-patient Specvis comparisons. Specvis calculates the dB range between the maximum and minimum stimulus luminance or the ratio of maximum stimulus luminance to the luminance threshold below which the stimulus is not perceived at a given location in dB based on the following formula:

$$\Delta L=\;10\;\times \;log_{10}\left(\frac{L_{max}}{L_{T}}\right)$$

where *L*_*max*_ = the maximum possible stimulus luminance in cd/m^2^ that can be used in the visual field examination; *L*_*T*_ = the minimum possible stimulus luminance in cd/m^2^, but also = the luminance threshold below which the stimulus is not perceived at a given location; Δ*L* = the ratio of *L*_*max*_ to *L*_*T*_ expressed in dB.

Regardless of the perimetry type used, stimuli are displayed on a hemispherical surface and the examined eye is positioned at the geometric center of this surface, so that all locations are equidistant from the eye. This is not the case with flat screens for which the elements are spread equally on the screen and distance expressed in pixels, but are not equally spread across the patient’s visual field. This is accomplished by turning on the *Correction for the sphericity of the field of view*, which results in spreading screen elements equally in relation to the patient’s visual field, and the distance between elements is expressed in degrees of arc. This functionality is based on the following formula:

$$D=\;\frac{x}{\text{tan}{\left(\beta \right)}^{-1}}$$

where the variable *x* = the distance in mm between the patient and screen; *β* = the distance in degrees of arc from the fixation point to the displayed element; *D* = a corrected distance in mm from the fixation point to the element. Because screen display elements using pixels it is necessary to convert mm to pixels according to the formula:

$$P=D\frac{R}{y}$$

where *y* = screen width or height in mm (depending on whether horizontal or vertical correction for sphericity is used); *R* = the screen resolution in pixels; *D* = a corrected distance in mm from the fixation point to the element; *P* = *D* expressed in pixels. Finally, the examiner can preview the distribution of all procedure elements (S6 Fig) and verify them.

### Adjusting fixation and other options

The last obligatory window before starting the visual field examination test contains adjustments for fixation and other options (Fig 12). Specvis allows for a basic setting of fixation point characteristics such that color, size and location on the screen can be adjusted making it possible to examine the whole visual field. However, it is necessary to emphasize, that the position of the fixation point should be always perpendicular to the position of the cornea of the tested eye. When examining both eyes simultaneously, the fixation point position should be perpendicular to the middle of a line connecting the corneas. Fixation point color is separated from stimuli and background luminance scales, therefore if it is necessary to know the luminance value of the fixation point of a given color, this must be measured individually.

Specvis utilizes three different methods for controlling patient fixation. One, called *Blindspot*, is similar to that used in the MM700, i.e. a false positive in response to presumed optic disc stimulation (the Heijl-Krakau technique). The user can modify the characteristics of the *control* stimulus (specific stimulus displayed in presumed blind spot location), such as size, brightness, presentation frequency, and the position on the screen (S7 Fig). The control stimulus shares the luminance scale with *ordinary* stimuli (displayed outside optic disc location). If the patient responds to the *control* stimulus, Specvis assumes that the eye has moved off the fixation point.

The second method for controlling patient fixation, called *Fixation point change*, is based on changes in the characteristics of the fixation point. During the visual field examination the appearance of the fixation point periodically changes in the domain of size and color. The patient’s task is to respond to the presented stimuli and to the fixation point changes, and a failure to respond to fixation point changes indicates that the eye has moved off the fixation point. The examiner can configure the appearance of the *changed* fixation point with respect to size, color, luminance, and frequency of changes in the appropriate window (S8 Fig).

The third option is called *Both* and it uses both: *Blindspot* and *Fixation point change* techniques in order to monitor the fixation accuracy. User can freely adjust options for each of the “partial” techniques.

Specvis also provides feedback messages to the patient when they lose fixation (S9 Fig). This function is available for both of the fixation control techniques. In the settings window for this function the user can change the displayed text, its location, color, and font size. When the examination is performed with dual screens, i.e. allowing the examiner to also view the test in progress, fixation loss information is also available to the examiner.

The keyboard configuration can also be changed and keys defined for three Specvis action. The patient should only use the *Answer to stimulus* button, while the *Pause procedure* and *Cancel procedure* should be used only by the examiner. Pause and resume functions have no effect on the data, however, the cancel function will cause the loss of all currently recorded data. In addition, using pause do not stop the counter of the test duration.

### Specvis visual field examination procedure

**Fig 14:**
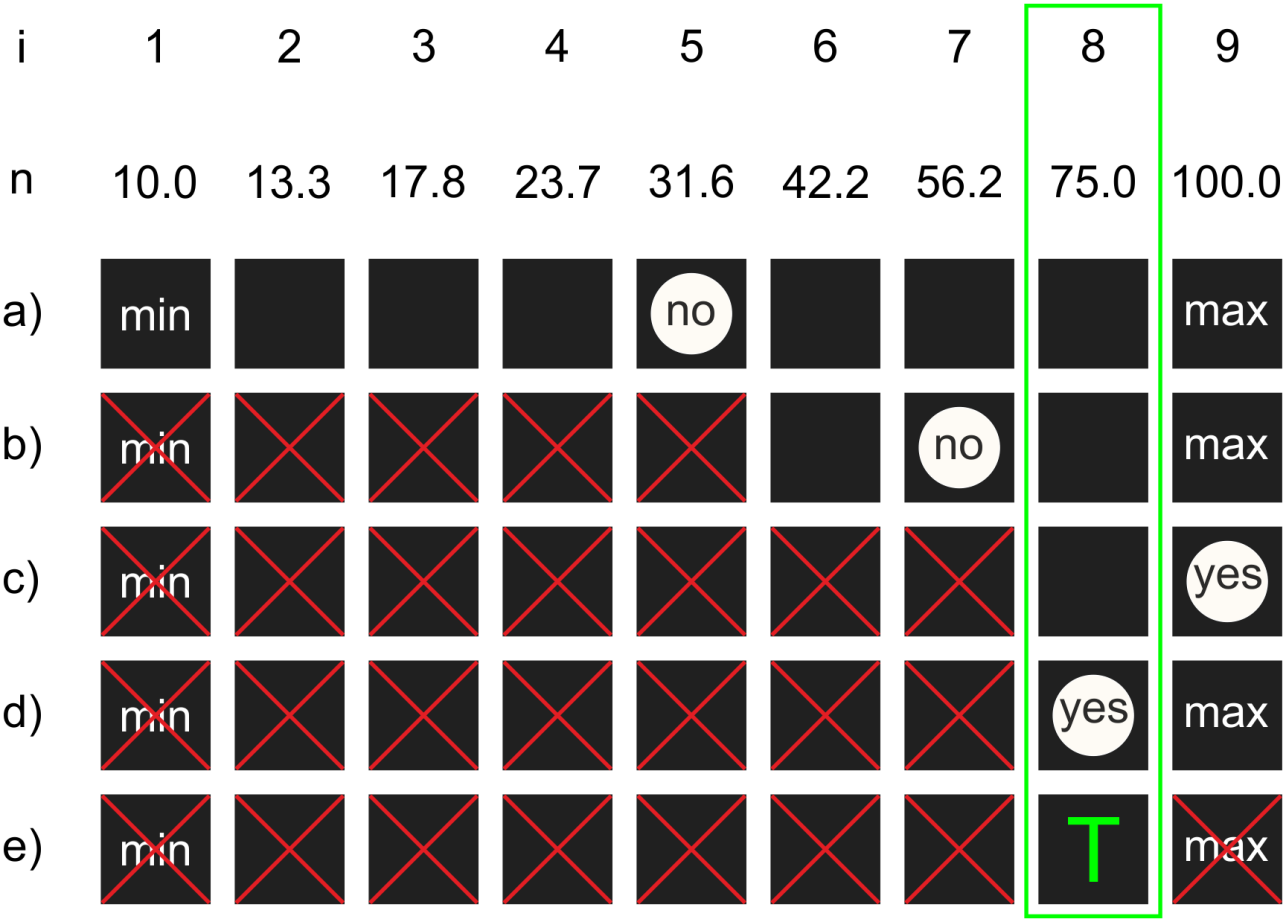
The scheme of the *Basic* procedure. During the initialization of the visual field test, Specvis creates a vector of *i* equally or logarithmically (optional) spaced *n* brightness values from the range defined by the minimum and maximum stimulus brightness. At each stimulus location, Specvis estimates a brightness threshold. An example threshold estimation for a given stimulus location can look like: a) display the stimulus with a brightness *i* = 5 and read the patient response; b) if the patient did not respond, discard brightness values from *i* = 1 to *i* = 5 inclusive and display the stimulus with a brightness *i* = 7; c) if the patient did not respond, discard brightness values from *i* = 6 to *i* = 7 inclusive and display the stimulus of brightness *i* = 9; d) if patient responded to the stimulus, display the stimulus with a brightness *i* = 8; e) if the patient responded, discard brightness value *i* = 9 and set value *i* = 8 (*n* = 75) as the estimated brightness threshold for a given stimulus location. Finally, find fitted luminance value for the threshold brightness value.

Finally, the examiner selects and runs the examination procedure. Currently, there is only one procedure i.e. *Basic* (Fig 14), which is a typical staircase procedure and presents stimuli in a random order at predefined locations on the screen. At each location, Specvis estimates the luminance threshold. The main purpose is to achieve a fast screening procedure designed to evaluate the general condition of the visual field. The user can modify two parameters of this procedure. It can set the length of the tested brightness vector, which holds the range of minimum and maximum stimulus brightness values that will be individually tested at all predefined locations. Specvis starts testing from the middle of this vector and moves along it by one or two values depending on the patient response until a luminance threshold is reached at a given location. Taking into account this stepping rule, the vector length must be of a specific value, i.e.:

5+4n

where *n* is a natural number, which gives 9, 13, 17, 21 etc. In the *Basic* procedure, the brightness vector values are spread equally but it is also possible to spread them logarithmically.

### Conducting visual field test and monitoring test progress

**Fig 15:**
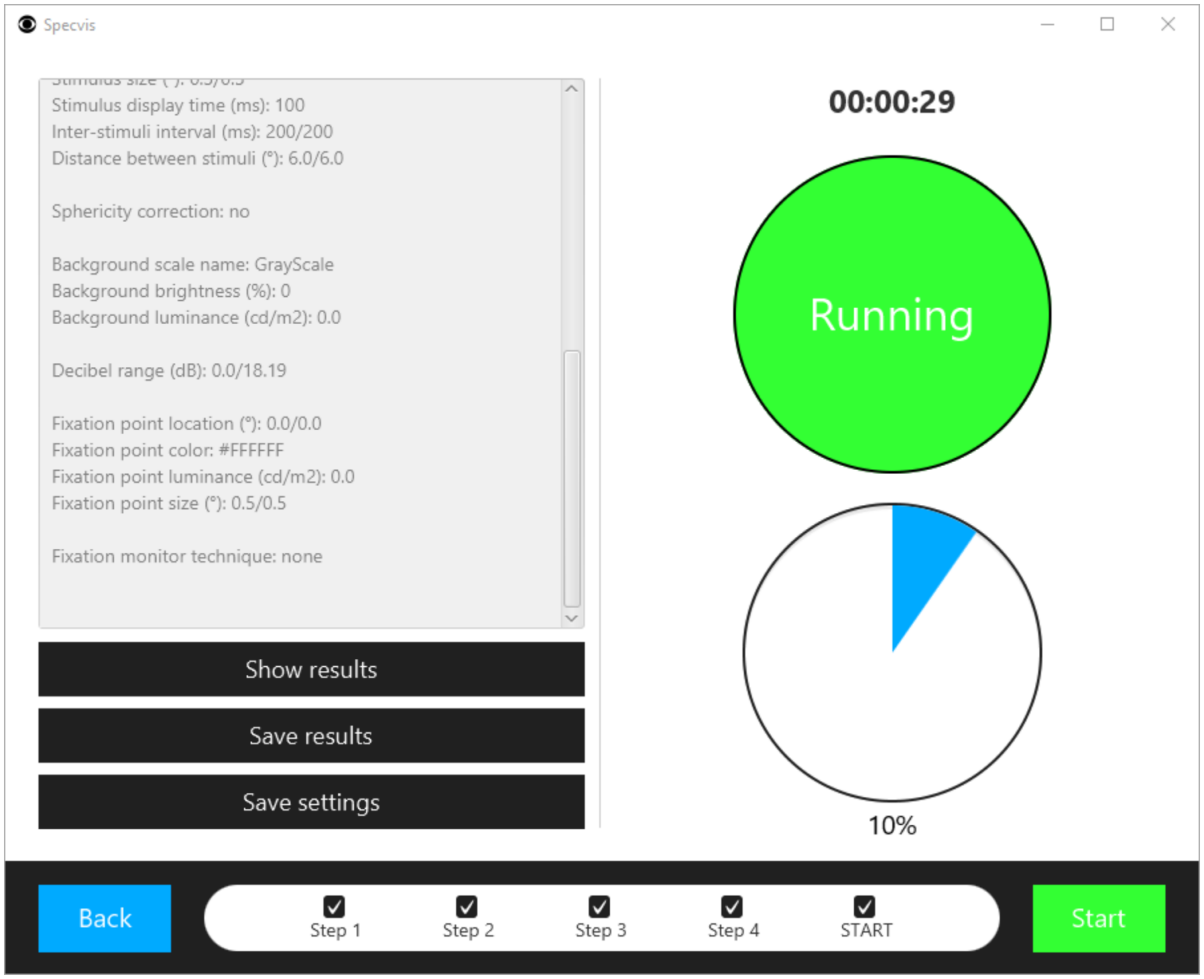
Window for monitoring the progress of the visual test. Before clicking the Start button, which will run the visual field test, the user can verify and edit all key parameters of the procedure in a scrollable text area. In the event that the user has access to two screens, it is possible (and recommended) to use one screen for displaying the visual field test and the other to monitor the progress of the test. When the test is finished, the user can show the results to the patient and then save them and the settings used in the test in the form of a *.sset file.

After setting all the necessary options, the user can move to the procedure window (Fig 15). We strongly recommend using two screens if possible while performing the examination, thus allowing the user to actively control and monitor the procedure without disturbing the patient as they view just the stimulus presentation.

During the test, Specvis calculates a false-positive response rate. This rate, along with information about fixation accuracy, may help the examiner to evaluate the reliability of the test and to indicate if repeated tests are needed. The false-positive response rate is calculated as follows:

$$FPRR=\;\frac{F}{F+P}\;\times \;100$$

where *F* = false positives (e.g. responding to a lack of stimulus); *P* = positive responses; *FPRR* = the false-positive response rate expressed as a percentage. The permission to respond to a given stimulus is always granted when the stimulus is presented on the screen, and ends with the patient response or the presentation of the next stimulus.

The results are saved in the *Results* directory in individual patient folders with the name of the patient’s ID. Each set of the results has its own dedicated folder with a randomly created name based on the template R_yyyyMMdd_xxxx, where the syntax is the same as that for the patient ID and luminance scale but with prefix R to denote these are results files.

In this procedure window it is also possible to save a Specvis settings template in a *.sset file format in *Settings* directory, so that it can be loaded during the next test from the level of the very first window that will be displayed to the user after the Specvis launch.

## Supporting information

S1 Fig Glaucomatous patient no. 1; MM700 and Specvis visual field graphical maps. Conventions are the same as in Fig 2.
(TIF)

S2 Fig Glaucomatous patient no. 2; MM700 and Specvis visual field graphical maps. Conventions are the same as in Fig 2.
(TIF)

S3 Fig Glaucomatous patient no. 3; MM700 and Specvis visual field graphical maps. Conventions are the same as in Fig 2.
(TIF)

S4 Fig Stroke patient no. 2; MM700 and Specvis visual field graphical maps. Conventions are the same as in Fig 3.
(TIF)

S5 Fig Window for creating new luminance scale. (TIF)

S6 Fig Distribution preview of all procedure elements. S = predefined stimulus location; F = predefined fixation point location; M = assumed blind spot location.
(TIF)

S7 Fig Window for adjusting *Blindspot* fixation monitor technique settings. (TIF)

S8 Fig Window for adjusting *Fixation point change* fixation monitor technique settings. (TIF)

S9 Fig Window for adjusting settings of message displayed to the patient after fixation loss. (TIF)

S10 Fig Window displaying the result of comparison of two chosen patient’s datasets. (TIF)

S1 Table Summary data for retinitis pigmentosa patient examined with Specvis. Each eye of the retinitis pigmentosa patient was tested three times.
(PDF)

S2 Table Summary data for stroke patients examined with Specvis. Each eye of the stroke patients was tested three times.
(PDF)

S3 Table Summary data for all control subjects from all tests for all groups each using different fixation monitor technique, i.e. *Blindspot*, *Fixation point change*, and *Both*. (PDF)

## References

[1] Weinreb RN, Aung T, Medeiros FA. The pathophysiology and treatment of glaucoma: a review. JAMA. 2014; 311 (18): 1901–11. doi: 10.1001/jama.2014.3192 24825645 10.1001/jama.2014.3192 PMC4523637

[2] Tham YC, Li X, Wong TY, Quigley HA, Aung T, Cheng CY. Global prevalence of glaucoma and projections of glaucoma burden through 2040: a systematic review and meta-analysis. Ophthalmology. 2014; 121 (11): 2081–90. doi: 10.1016/j.ophtha.2014.05.013 24974815 10.1016/j.ophtha.2014.05.013

[3] Lim LS, Mitchell P, Seddon JM, Holz FG, Wong TY. Age-related macular degeneration. Lancet. 2012; 379 (9827): 1728–38. doi: 10.1016/S0140-6736(12)60282-7 22559899 10.1016/S0140-6736(12)60282-7

[4] Ciulla TA, Amador AG, Zinman B. Diabetic retinopathy and diabetic macular edema: pathophysiology, screening, and novel therapies. Diabetes Care. 2003; 26 (9); 2653–64. 12941734 10.2337/diacare.26.9.2653

[5] Duh E. Diabetic retinopathy. Humana Press; 2008.

[6] Yau JW, Rogers SL, Kawasaki R, Lamoureux EL, Kowalski JW, Bek T, et al Global prevalence and major risk factors of diabetic retinopathy. Diabetes Care. 2012; 35 (3): 556–64. doi: 10.2337/dc11-1909 22301125 10.2337/dc11-1909 PMC3322721

[7] Aldrich MS, Alessi AG, Beck RW, Gilman S. Cortical blindness: etiology, diagnosis, and prognosis. Ann Neurol. 1987; 21 (2): 149–58. doi: 10.1002/ana.410210207 3827223 10.1002/ana.410210207

[8] Gaber TA. Rehabilitation of cortical blindness secondary to stroke. NeuroRehabilitation. 2010; 27 (4): 321–5. doi: 10.3233/NRE-2010-0615 21160121 10.3233/NRE-2010-0615

[9] Taylor H, Keeffe J. World blidness: a 21st century perspective. Br J Ophthalmol. 2001; 85 (3): 261–266. doi: 10.1136/bjo.85.3.261 11222327 10.1136/bjo.85.3.261 PMC1723903

[10] Resnikoff S, Pascolini D, Etya’ale D, Kocur I, Pararajasegaram R, Pokharel GP, et al Global data on visual impairment in the year 2002. Bull World Health Organ. 2004; 82 (11): 844–51. 15640920 PMC2623053

[11] World Health Organization. Global data on visual impairments 2010. WHO/NMH/PBD/12.01. Accessed: http://www.who.int/blindness/GLOBALDATAFINALforweb.pdf.

[12] Gordois A, Cutler H, Pezzullo L, Gordon K, Cruess A, Winyard S, et al As estimation of the worldwide economic and health burder of visual impairment. Glob Public Health. 2012; 7 (5): 465–81. doi: 10.1080/17441692.2011.634815 22136197 10.1080/17441692.2011.634815

[13] Pascolini D, Mariotti SP. Global estimates of visual impairment: 2010. Br J Ophthalmol. 2012; 96 (5): 614–8. doi: 10.1136/bjophthalmol-2011-300539 22133988 10.1136/bjophthalmol-2011-300539

[14] Thrift AG, Cadilhac DA, Thayabaranathan T, Howard G, Howard VJ, Rothwell PM, et al Global stroke statistics. Int J Stroke. 2014; 9 (1): 6–18. doi: 10.1111/ijs.12245 24350870 10.1111/ijs.12245

[15] Antal A, Paulus W, Nitsche MA. Electrical stimulation and visual network plasticity. Restor Neurol Neurosci. 2011; 29 (6): 365–74. doi: 10.3233/RNN-2011-0609 22124032 10.3233/RNN-2011-0609

[16] Fedorov A, Jobke S, Bersnev V, Chibisova A, Chibisova Y, Gall C, et al Restoration of vision after optic nerve lesions with noninvasive transorbital alternating current stimulation: a clinical observational study. Brain Stimul. 2011; 4 (4): 189–201. doi: 10.1016/j.brs.2011.07.007 21981854 10.1016/j.brs.2011.07.007

[17] Gall C, Silvennoinen K, Granata G, de Rossi F, Vecchio F, Brösel D, et al Non-invasive electric current stimulation for restoration of vision after unilateral occipital stroke. Contemp Clin Trials. 2015; 43: 231–6. doi: 10.1016/j.cct.2015.06.005 26072125 10.1016/j.cct.2015.06.005

[18] Gekeler F, Zrenner E, Bartz-Schmidt KU. Ocular electrical stimulation: Therapeutic application and active retinal implants for hereditary retinal degenerations. Ophthalmologe. 2015; 112 (9): 712–9. doi: 10.1007/s00347-015-0126-3 26319085 10.1007/s00347-015-0126-3

[19] Tropepe V, Coles BL, Chiason BJ, Horsford DJ, Elia AJ, McInnes RR, et al Retinal stem cells in the adult mammalian eye. Science. 2000; 287 (5460): 2032–6. 10720333 10.1126/science.287.5460.2032

[20] Ramsden CM, Powner MB, Carr AJ, Smart MJ, da Cruz L, Coffey PJ. Stem cells in retinal regeneration: past, present and future. Development. 2013; 140 (12): 2576–85. doi: 10.1242/dev.092270 23715550 10.1242/dev.092270 PMC3666384

[21] Rolling F. Recombinant AAV-mediated gene transfer to the retina: gene therapy perspectives. Gene Ther. 2004; 11 Suppl 1: 26–32. 10.1038/sj.gt.3302366 15454954

[22] Elliott DB, North I, Flanagan J. Confrontation visual field tests. Ophthalmic Physiol Opt. 1997; 17 Suppl 2: 17–24. 10.1016/s0275-5408(97)00045-8 9666904

[23] Aulhorn E, Harms H. Chapter 5: Visual perimetry In: Jameson D, Hurvich LM, Alpern M, editors. Visual psychophysics. Springer-Verlag; 1972 pp. 102–144.

[24] Delgado MF, Nguyen NT, Cox TA, Singh K, Lee DA, Dueker DK, et al Automated perimetry: a report by the American Academy of Ophthalmology. Ophthalmology. 2002; 109 (12): 2362–74. 12466186 10.1016/s0161-6420(02)01726-8

[25] Schiefer U, Pätzold J, Dannheim F. Conventional perimetry I: introduction—basics. Ophthalmologe. 2005; 102 (6): 627–44. 15906041 10.1007/s00347-005-1189-3

[26] Wójcik-Gryciuk A, Skup M, Waleszczyk WJ. Glaucoma—state of the art and perspectives on treatment. Restor Neurol Neurosci. 2015; 34 (1): 107–23. doi: 10.3233/RNN-150599 26684267 10.3233/RNN-150599 PMC4927811

[27] Johansson GA, Fitzmorris TK, Patella VM. Systems and methods for improved visual field testing. United States Patent. Patent no. US8684529B2. 2014.

[28] Burke W, Dreher B, Wang C. Selective block of conduction in Y optic nerve fibres: significance for the concept of parallel processing. Eur J Neurosci. 1998; 10 (1): 8–19. 9753109 10.1046/j.1460-9568.1998.00025.x

[29] Wang C, Waleszczyk WJ, Benedek G, Burke W, Dreher B. Convergence of Y and non-Y channels onto single neurons in the superior colliculi of the cat. Neuroreport. 2001; 12 (13): 2927–33. 11588605 10.1097/00001756-200109170-00035

[30] Waleszczyk WJ, Wang C, Young JM, Burke W, Calford MB, et al Laminar differences in plasticity in area 17 following retinal lesions in kittens or adult cats. Eur J Neurosci. 2003; 17 (11): 2351–68. 12814367 10.1046/j.1460-9568.2003.02674.x

[31] Waleszczyk WJ, Wang C, Benedek G, Burke W, Dreher B. Motion sensitivity in cat’s superior colliculus: contribution of different visual processing channels to response properties of collicular neurons. Acta Neurobiol Exp (Wars). 2004; 64 (2): 209–28. 15366254 10.55782/ane-2004-1507

[32] Yuan N, Liang Z, Yang Y, Li G, Zhou Y. Changes of spatial and temporal frequency tuning properties of neurons in the middle temporal area of aged rhesus monkeys. Eur J Neurosci. 2014; 40 (4): 2652–61. doi: 10.1111/ejn.12634 24888415 10.1111/ejn.12634

[33] Bola M, Gall C, Sabel BA. The second face of blindness: processing speed deficits in the intact visual field after pre- and post-chiasmatic lesions. PLoS One. 2013; 8 (5): e63700 doi: 10.1371/journal.pone.0063700 23667657 10.1371/journal.pone.0063700 PMC3648511

[34] Barton JJ, Rizzo M. Motion perception in optic neuropathy. Neurology. 1994; 44 (2): 273–8. 8309573 10.1212/wnl.44.2.273

[35] Raz N, Dotan S, Benoliel T, Chokron S, Ben-Hur T, Levin N. Sustained motion perception deficit following optic neuritis: Behavioral and cortical evidence. Neurology. 2011; 76 (24): 2103–11. doi: 10.1212/WNL.0b013e31821f4602 21670440 10.1212/WNL.0b013e31821f4602

[36] Raz N, Dotan S, Chokron S, Ben-Hur T, Levin N. Demyelination affects temporal aspects of perception: an optic neuritis study. Ann Neurol. 2012; 71 (4): 531–8. doi: 10.1002/ana.22692 22447670 10.1002/ana.22692

[37] Yu-Wai-Man P, Chinnery PF. Leber hereditary optic neuropathy GeneReviews. Accessed: https://www.ncbi.nlm.nih.gov/books/NBK1174/. 10.1136/jmg.39.3.162 PMC1735056 11897814

[38] Gosling J, Joy B, Steele G, Bracha G, Buckley A. The Java Language Specification Java SE 8 Edition Accessed: https://docs.oracle.com/javase/specs/jls/se8/jls8.pdf.

[39] Eckel B. Thinking in Java. Prentice Hall; 2006.

[40] Free Software Foundation. GNU Operating System. GNU General Public License version 3. Accessed: http://www.gnu.org/licenses/gpl.txt.

