# Supplementary figures and images for: Specvis: Free and open-source software for visual field examination
Source: PLoS One. 2017 Oct 13;12(10):e0186224. doi: 10.1371/journal.pone.0186224 (PMC5640235; doi:10.1371/journal.pone.0186224)

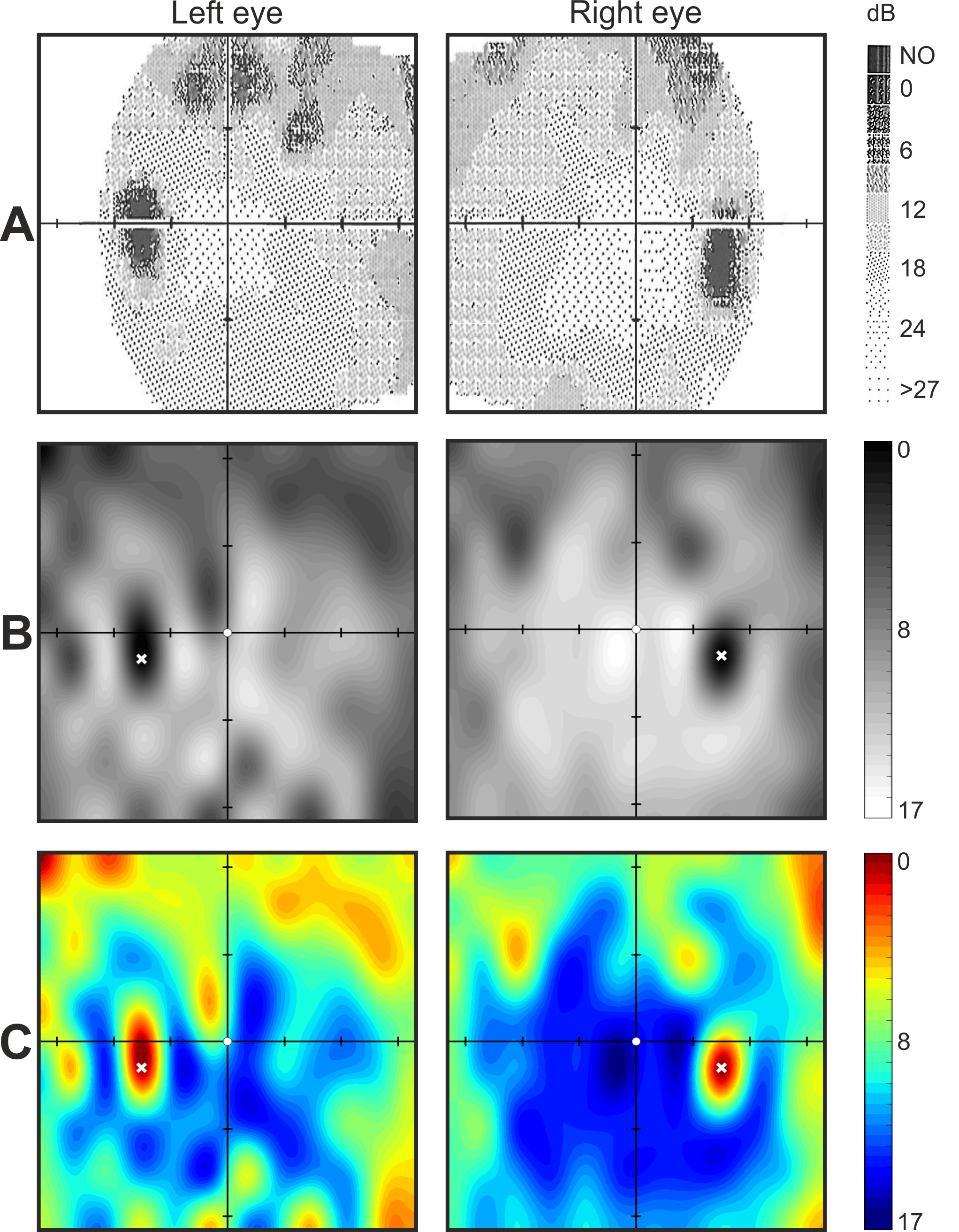

Supplement: S1 Fig — Conventions are the same as in Fig 2. (TIF) [file pone.0186224.s001.tif]

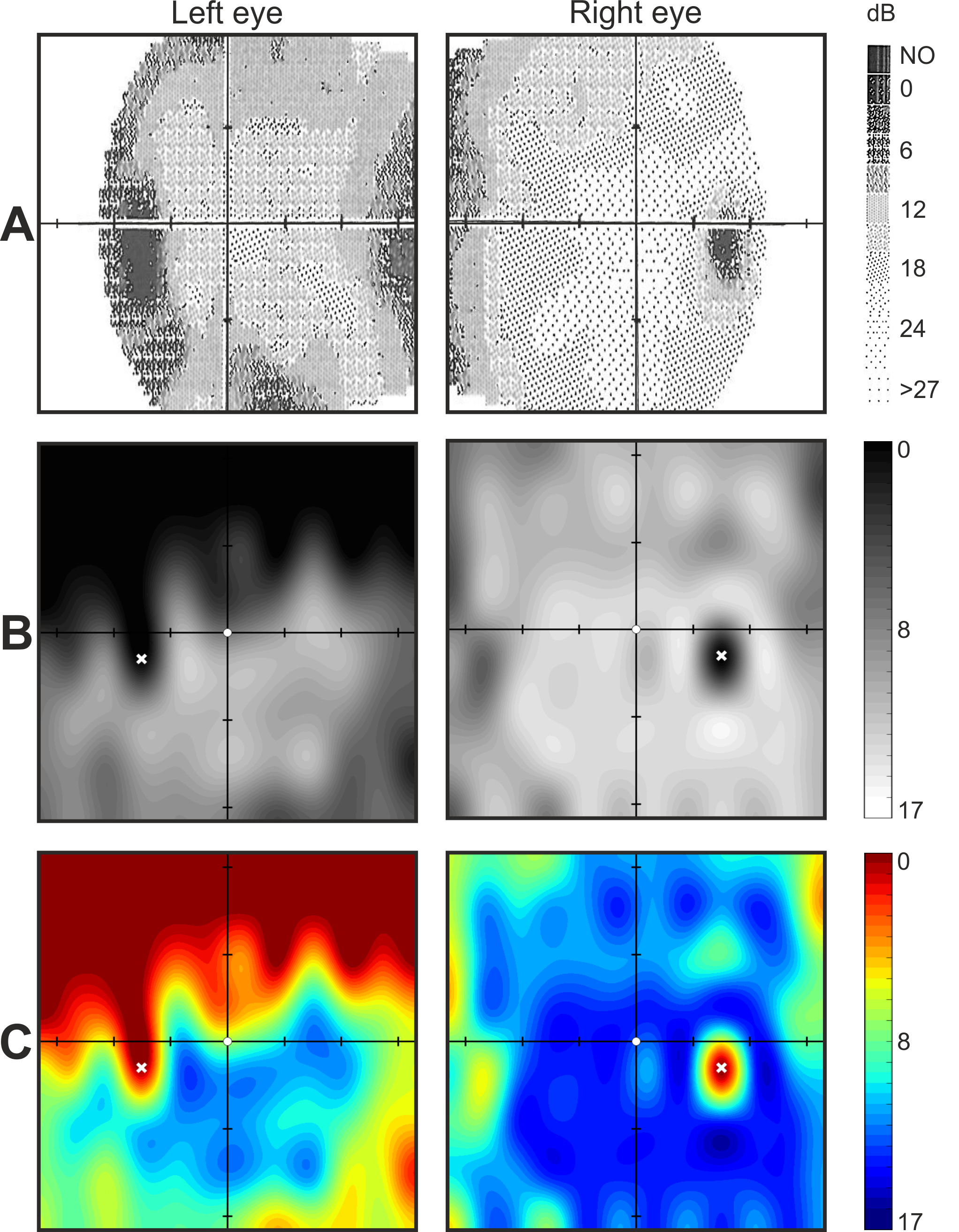

Supplement: S2 Fig — Conventions are the same as in Fig 2. (TIF) [file pone.0186224.s002.tif]

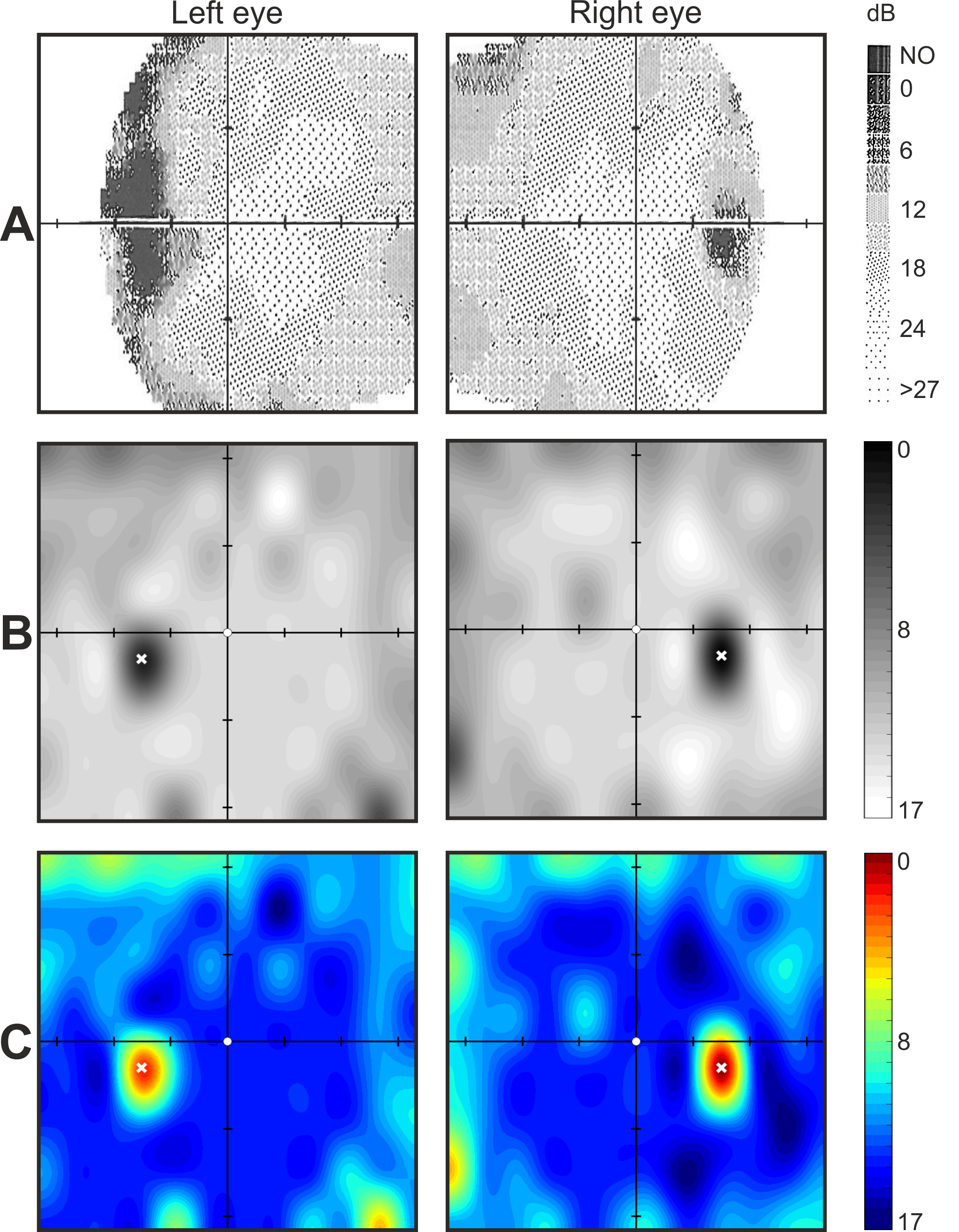

Supplement: S3 Fig — Conventions are the same as in Fig 2. (TIF) [file pone.0186224.s003.tif]

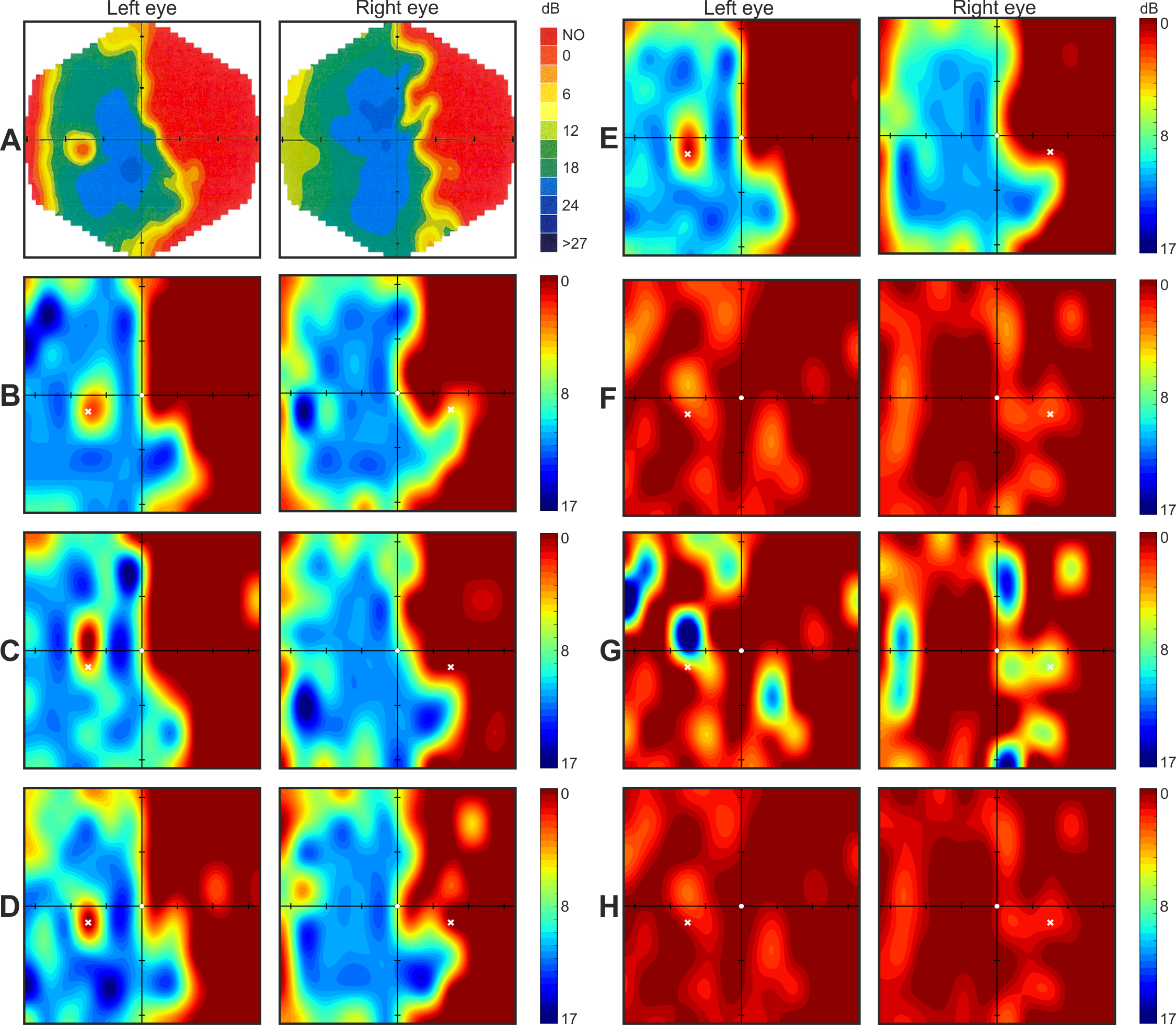

Supplement: S4 Fig — Conventions are the same as in Fig 3. (TIF) [file pone.0186224.s004.tif]

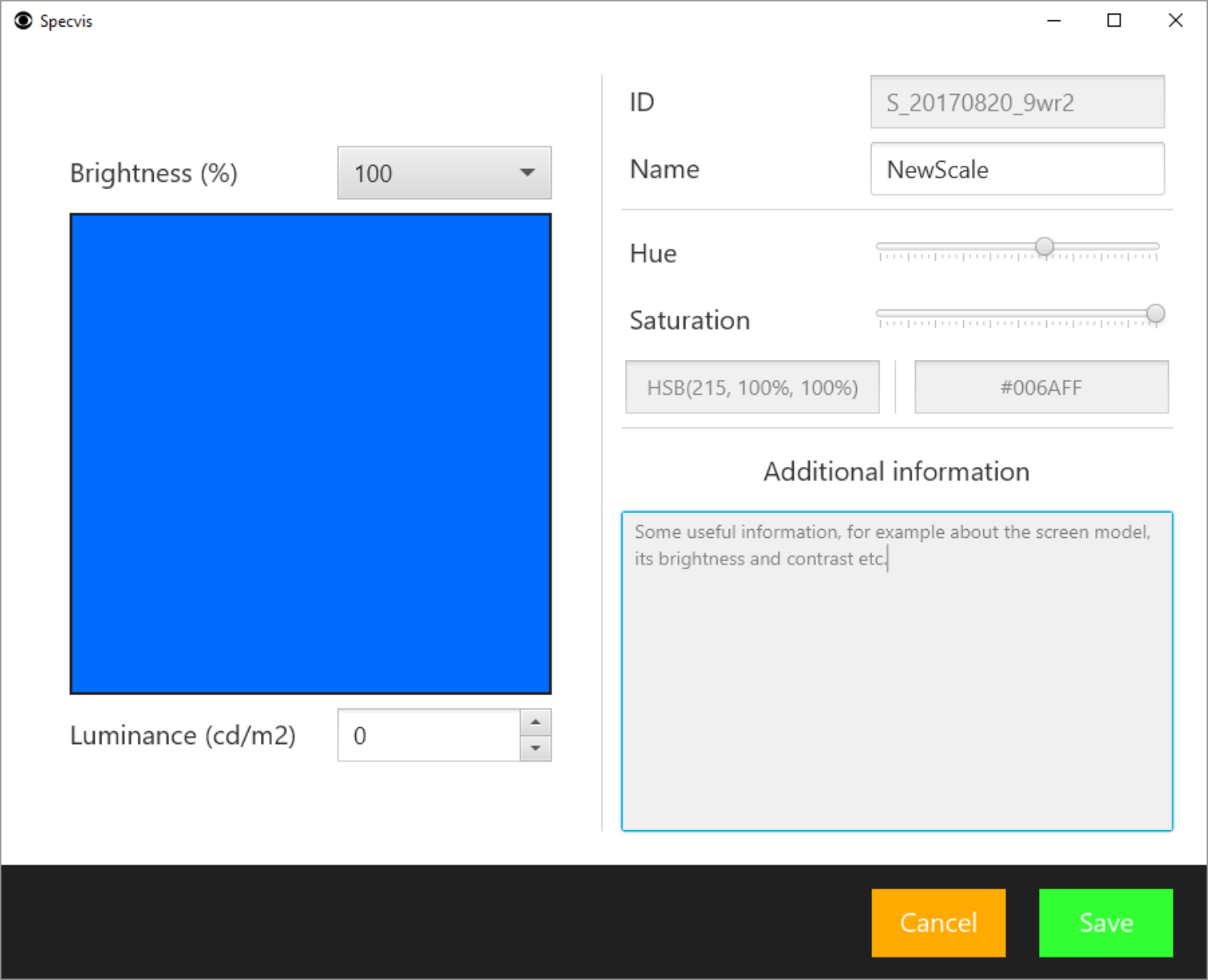

Supplement: S5 Fig — (TIF) [file pone.0186224.s005.tif]

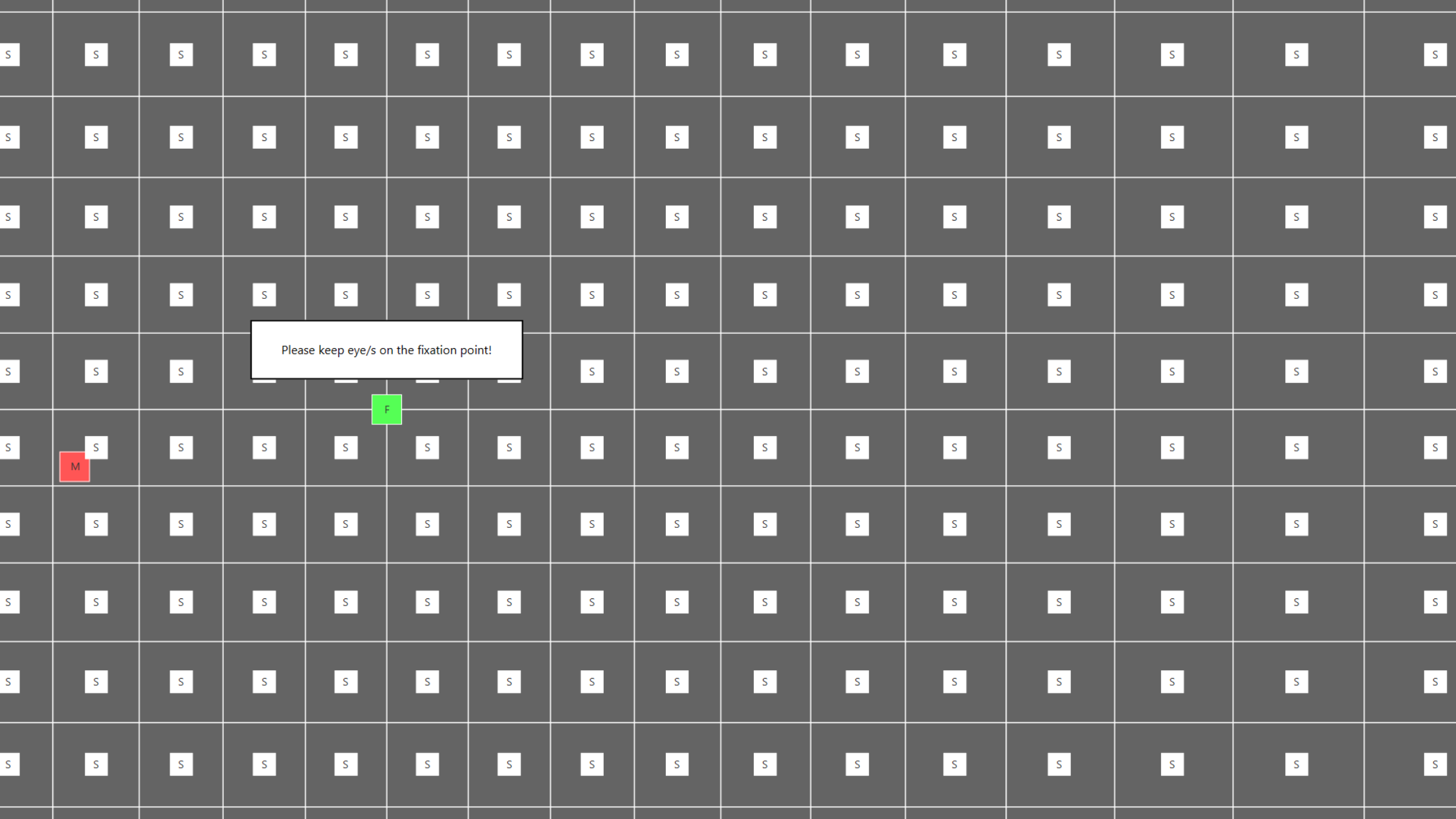

Supplement: S6 Fig — S = predefined stimulus location; F = predefined fixation point location; M = assumed blind spot location. (TIF) [file pone.0186224.s006.tif]

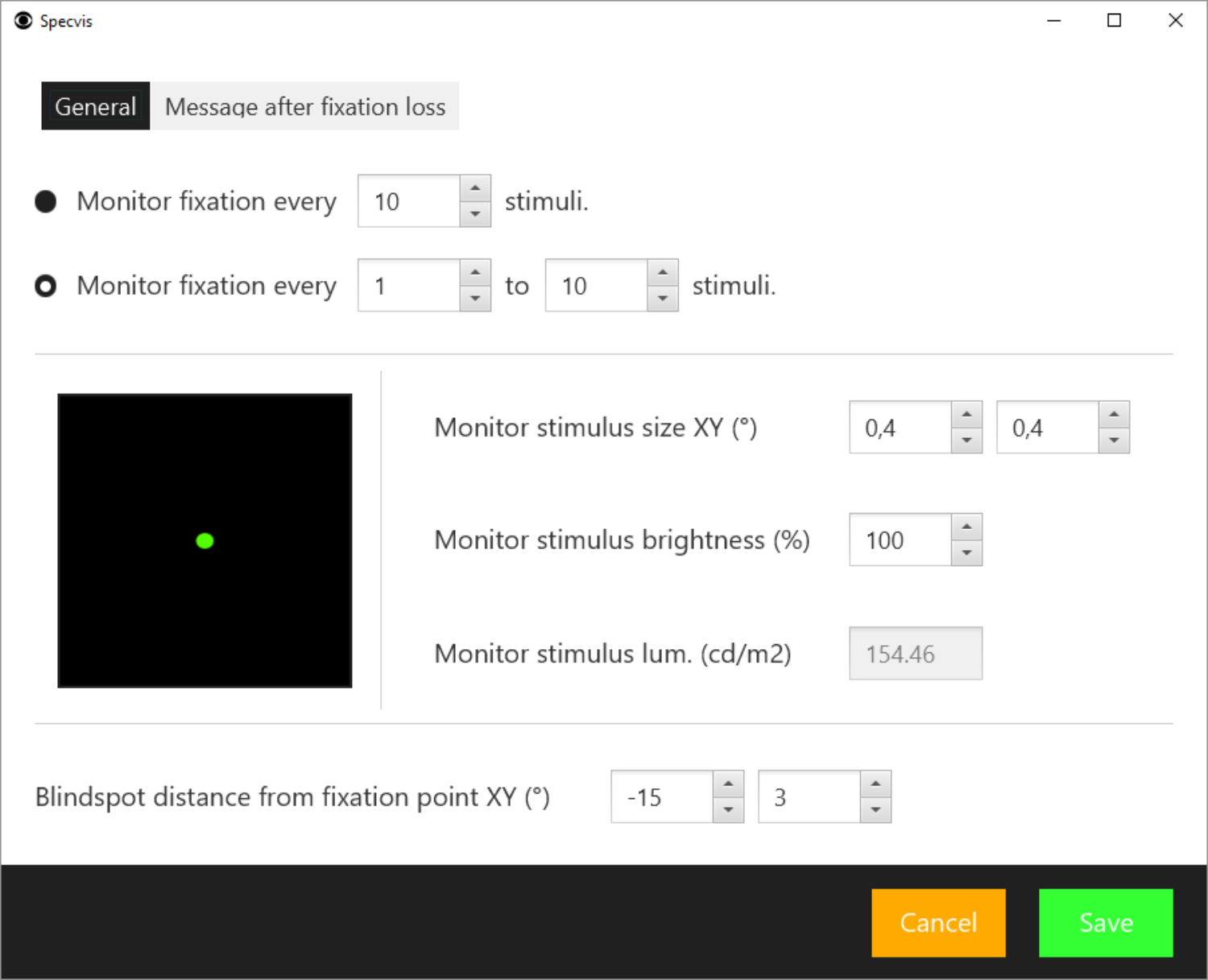

Supplement: S7 Fig — (TIF) [file pone.0186224.s007.tif]

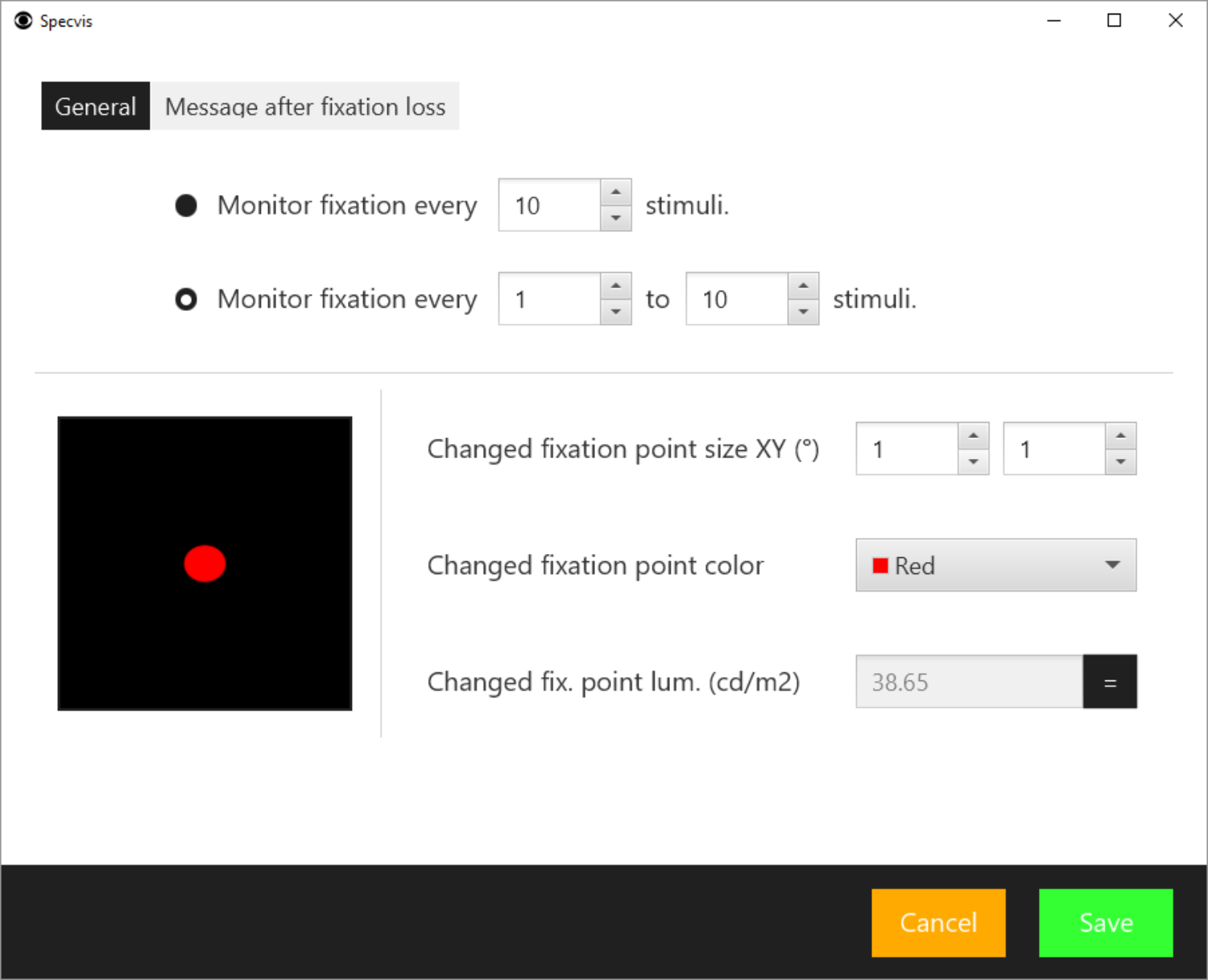

Supplement: S8 Fig — (TIF) [file pone.0186224.s008.tif]

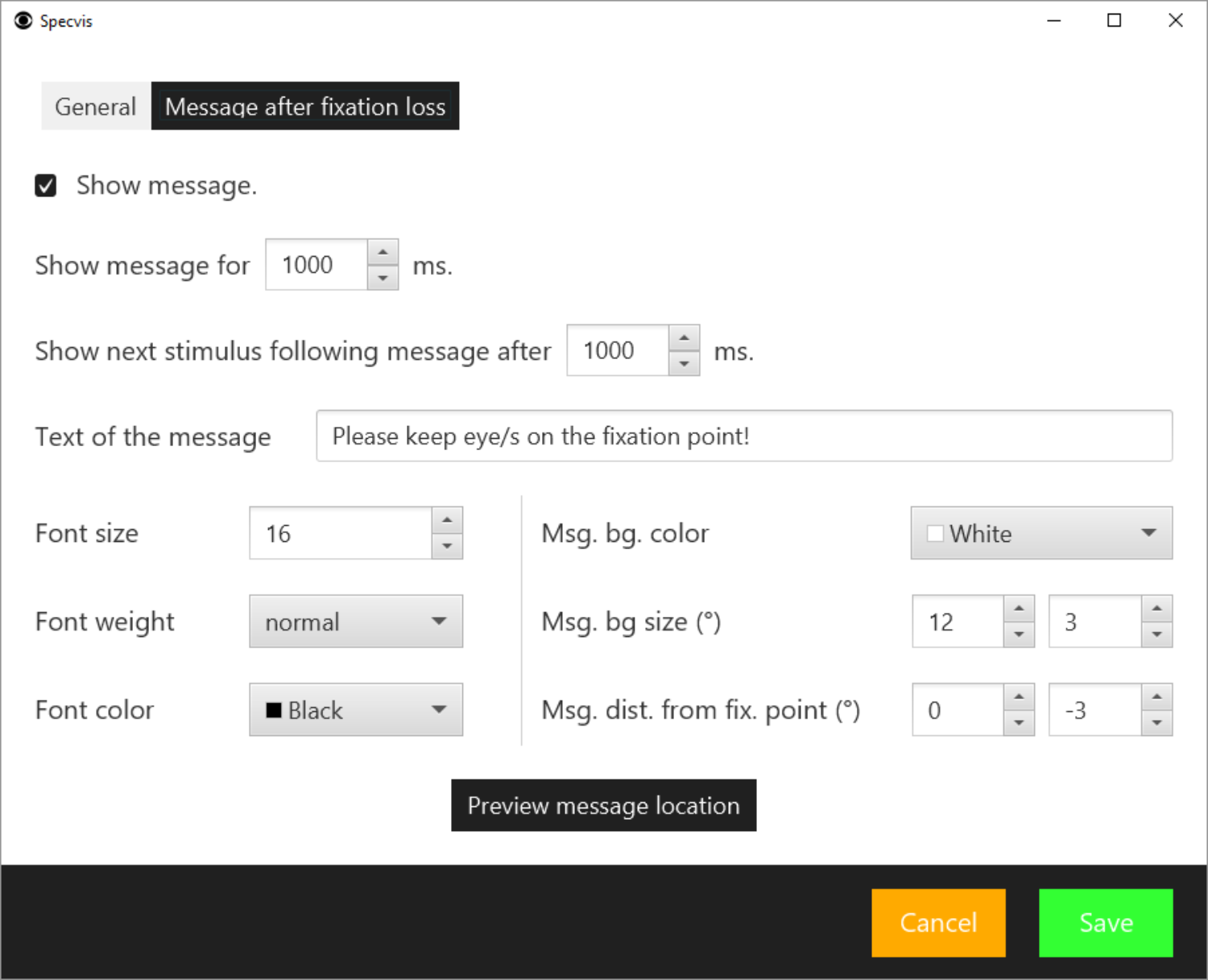

Supplement: S9 Fig — (TIF) [file pone.0186224.s009.tif]

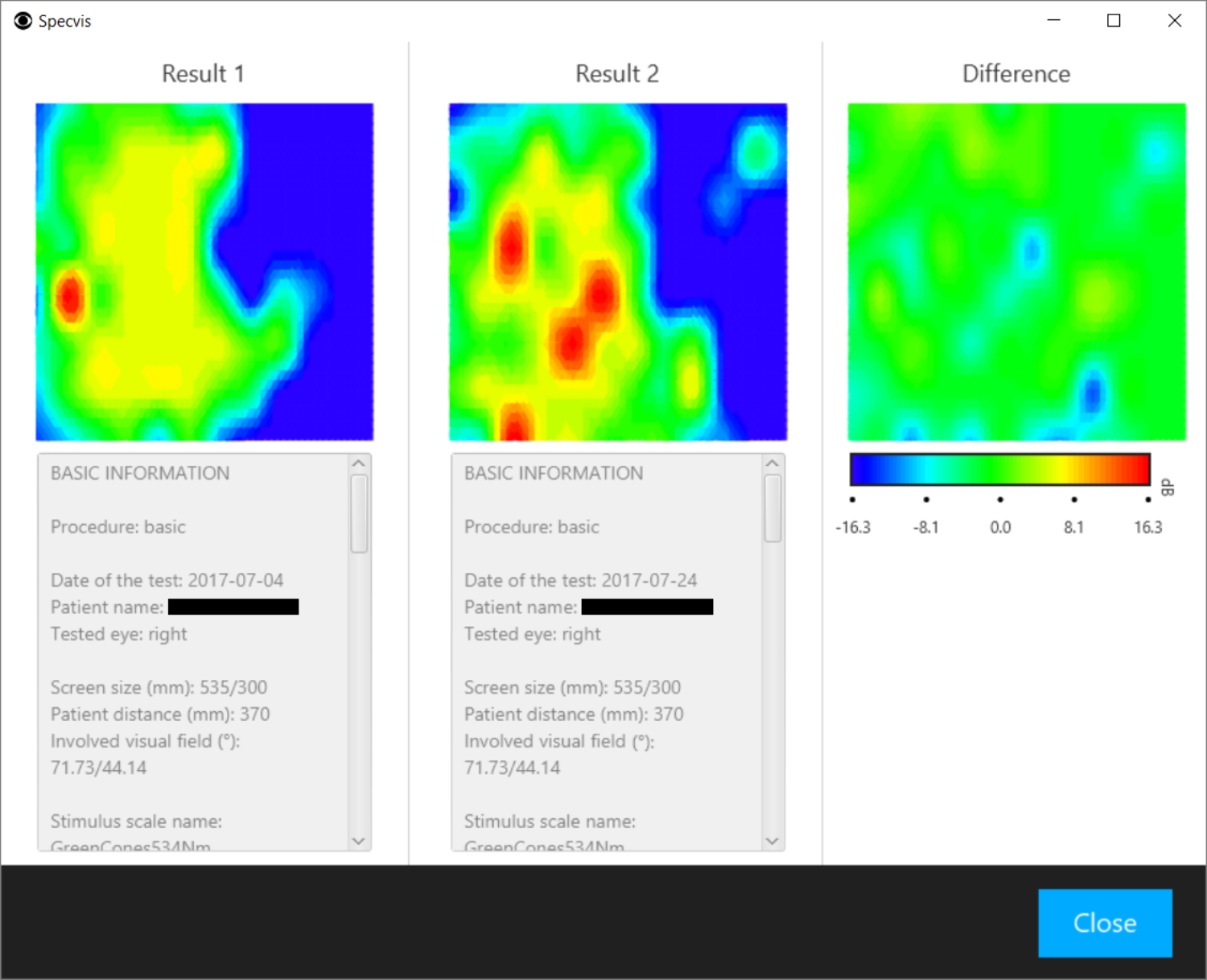

Supplement: S10 Fig — (TIF) [file pone.0186224.s010.tif]
