# Supplementary material for: Specvis: Free and open-source software for visual field examination
Source: PLoS One. 2017 Oct 13;12(10):e0186224. doi: 10.1371/journal.pone.0186224 (PMC5640235; doi:10.1371/journal.pone.0186224)
Supplement: S1 Table — Each eye of the retinitis pigmentosa patient was tested three times. (PDF) [file pone.0186224.s011.pdf]

S1 Table. Summary data for RP patient examined with Specvis. Each eye of the RP patient was tested three times.

Test	Eye	Duration	FA*	FA**	FPRR
1	Left	09:00.0	19/19 (100)	19/19 (100)	0/52 (0)
	Right	08:57.0	19/19 (100)	18/19 (95)	0/46 (0)
2	Left	09:04.0	20/20 (100)	20/20 (100)	0/49 (0)
	Right	09:11.0	21/21 (100)	20/20 (100)	0/49 (0)
3	Left	09:01.0	20/20 (100)	19/19 (100)	0/47 (0)
	Right	09:07.0	20/20 (100)	19/19 (100)	0/50 (0)
**Average**		09:03.3	19.8/19.8 (100.0)	19.2/19.3 (99.2)	0.0/48.8 (0.0)
**SD**		00:05.1	0.7/0.7 (0.0)	0.7/0.5 (1.9)	0.0/2.0 (0.0)

Conventions are the same as in the Table 2 and 4.
