# Supplementary material for: Specvis: Free and open-source software for visual field examination
Source: PLoS One. 2017 Oct 13;12(10):e0186224. doi: 10.1371/journal.pone.0186224 (PMC5640235; doi:10.1371/journal.pone.0186224)
Supplement: S2 Table — Each eye of the stroke patients was tested three times. (PDF) [file pone.0186224.s012.pdf]

S2 Table. Summary data for stroke patients examined with Specvis. Each eye of the stroke patient was tested three times.

Patient	Test	Eye	Duration	FA*	FA**	FPRR
1	1	Left	10:16.0	23/23 (100)	23/23 (100)	0/158 (0)
		Right	09:49.0	23/23 (100)	23/23 (100)	0/141 (0)
	2	Left	10:14.0	23/23 (100)	22/22 (100)	1/161 (1)
		Right	10:09.0	21/22 (95)	22/22 (100)	1/142 (1)
	3	Left	10:16.0	23/23 (100)	23/23 (100)	3/144 (2)
		Right	09:55.0	22/22 (100)	22/22 (100)	5/139 (3)
2	1	Left	10:24.0	14/23 (61)	22/22 (100)	4/181 (2)
		Right	10:12.0	20/22 (91)	21/22 (95)	18/158 (10)
	2	Left	10:42.0	5/23 (22)	21/23 (91)	5/175 (3)
		Right	10:20.0	21/23 (91)	22/22 (100)	52/163 (24)
	3	Left	10:09.0	14/21 (67)	19/21 (90)	143/158 (48)
		Right	09:59.0	22/23 (96)	21/21 (100)	77/162 (32)
Average			10:12.1	19.2/22.6 (85.2)	21.8/22.2 (98.0)	25.8/156.8 (10.5)
SD			00:14.0	5.3/0.6 (22.9)	1.1/0.7 (3.6)	42.4/12.8 (15.0)

Conventions are the same as in the Table 2 and 4.
