# Supplementary material for: Specvis: Free and open-source software for visual field examination
Source: PLoS One. 2017 Oct 13;12(10):e0186224. doi: 10.1371/journal.pone.0186224 (PMC5640235; doi:10.1371/journal.pone.0186224)
Supplement: S3 Table — (PDF) [file pone.0186224.s013.pdf]

S3 Table. Summary data for all subjects from all tests for all groups each using different fixation monitor technique, i.e. *Blindspot*, *Fixation point change*, and *Both*.

Subject	Test	*Blindspot*			Subject	*Fixation point change*			Subject	*Both*			
		Duration	FA	FPRR		Duration	FA	FPRR		Duration	FA*	FA**	FPRR
1	1	10:10.0	47/49 (96)	0/284 (0)	8	10:10.0	46/47 (98)	3/328 (1)	15	11:21.0	17/25 (68)	24/25 (96)	0/300 (0)
	2	10:19.0	50/50 (100)	0/281 (0)		10:08.0	50/50 (100)	1/334 (0)		11:14.0	17/24 (71)	23/23 (100)	0/302 (0)
	3	10:03.0	50/50 (100)	1/282 (0)		09:33.0	42/42 (100)	2/321 (1)		11:38.0	8/25 (32)	25/25 (100)	4/317 (1)
	4	10:04.0	46/48 (96)	0/286 (0)		11:57.0	43/43 (100)	2/322 (1)		11:48.0	13/24 (54)	23/23 (100)	0/312 (0)
	5	10:10.0	48/50 (96)	1/278 (0)		09:37.0	43/44 (98)	4/321 (1)		11:43.0	8/25 (32)	24/24 (100)	5/315 (2)
	6	10:04.0	47/49 (96)	0/287 (0)		10:40.0	39/40 (98)	3/322 (1)		11:27.0	5/24 (21)	23/23 (100)	0/307 (0)
2	1	09:45.0	45/47 (96)	1/247 (0)	9	10:19.0	51/51 (100)	0/329 (0)	16	11:01.0	23/24 (96)	23/23 (100)	0/303 (0)
	2	10:03.0	47/48 (98)	0/260 (0)		10:16.0	48/48 (100)	2/323 (1)		11:24.0	23/24 (96)	23/23 (100)	1/305 (0)
	3	09:51.0	43/46 (93)	0/251 (0)		10:15.0	50/50 (100)	0/323 (0)		11:14.0	24/24 (100)	24/24 (100)	0/306 (0)
	4	09:48.0	46/46 (100)	0/258 (0)		10:20.0	49/50 (98)	1/322 (0)		11:09.0	23/24 (96)	22/22 (100)	1/306 (0)
	5	09:53.0	46/47 (98)	0/248 (0)		10:00.0	46/46 (100)	0/324 (0)		11:25.0	25/25 (100)	24/24 (100)	1/307 (0)
	6	09:59.0	46/47 (98)	0/248 (0)		10:03.0	51/51 (100)	0/327 (0)		11:05.0	22/23 (96)	22/22 (100)	2/305 (1)
3	1	10:42.0	49/49 (100)	1/268 (0)	10	11:41.0	45/48 (94)	49/322 (13)	17	10:54.0	23/24 (96)	23/23 (100)	1/307 (0)
	2	10:19.0	49/51 (96)	0/265 (0)		10:00.0	47/48 (98)	3/322 (1)		11:10.0	22/25 (88)	24/24 (100)	0/310 (0)
	3	10:12.0	49/50 (98)	0/272 (0)		09:43.0	46/46 (100)	2/324 (1)		11:07.0	25/31 (81)	24/24 (100)	5/313 (2)
	4	10:08.0	48/48 (100)	2/266 (1)		10:08.0	43/45 (96)	111/317 (26)		11:06.0	23/23 (100)	22/22 (100)	0/305 (0)
	5	10:04.0	44/47 (94)	0/261 (0)		09:44.0	46/48 (96)	207/316 (40)		11:16.0	19/25 (76)	23/24 (96)	1/311 (0)
	6	10:01.0	50/50 (100)	0/267 (0)		09:55.0	47/47 (100)	5/321 (2)		10:52.0	19/23 (83)	22/22 (100)	0/307 (0)
4	1	10:25.0	46/50 (92)	14/265 (5)	11	10:18.0	52/52 (100)	3/333 (1)	18	11:18.0	20/24 (83)	23/24 (96)	1/282 (0)
	2	10:20.0	46/48 (96)	1/270 (0)		10:12.0	49/50 (98)	2/326 (1)		12:54.0	19/25 (76)	24/24 (100)	5/273 (2)
	3	10:16.0	49/50 (98)	1/262 (0)		11:40.0	50/51 (98)	0/320 (0)		11:08.0	23/26 (88)	25/26 (96)	7/277 (2)
	4	10:29.0	48/51 (94)	0/279 (0)		10:09.0	48/48 (100)	4/323 (1)		11:19.0	20/24 (83)	24/24 (100)	0/272 (0)
	5	10:34.0	49/52 (94)	6/268 (2)		10:26.0	45/48 (94)	0/316 (0)		11:23.0	21/25 (84)	22/22 (100)	3/280 (1)
	6	10:24.0	51/52 (98)	0/262 (0)		10:11.0	48/49 (98)	3/318 (1)		10:48.0	17/24 (71)	23/23 (100)	247/238 (51)
5	1	10:19.0	44/45 (98)	0/274 (0)	12	10:14.0	47/47 (100)	0/327 (0)	19	11:02.0	20/23 (87)	23/23 (100)	5/296 (2)
	2	09:56.0	44/46 (96)	0/278 (0)		10:28.0	50/51 (98)	0/328 (0)		11:19.0	11/24 (46)	23/24 (96)	8/311 (3)
	3	10:07.0	48/49 (98)	0/274 (0)		10:10.0	48/48 (100)	1/326 (0)		11:16.0	10/24 (42)	23/23 (100)	7/314 (2)
	4	10:11.0	47/49 (96)	0/270 (0)		10:17.0	52/52 (100)	1/327 (0)		11:17.0	13/24 (54)	24/24 (100)	2/298 (1)
	5	09:52.0	46/47 (98)	0/272 (0)		10:15.0	49/49 (100)	0/325 (0)		11:14.0	12/23 (52)	21/23 (91)	1/300 (0)
	6	10:07.0	48/50 (96)	1/276 (0)		10:07.0	44/45 (98)	0/318 (0)		11:19.0	14/25 (56)	23/23 (100)	4/308 (1)
6	1	10:50.0	41/49 (84)	4/278 (1)	13	10:01.0	48/48 (100)	0/288 (0)	20	11:05.0	23/24 (96)	22/23 (96)	0/299 (0)
	2	10:04.0	47/48 (98)	1/279 (0)		10:05.0	49/49 (100)	0/291 (0)		10:39.0	23/24 (96)	22/22 (100)	0/305 (0)
	3	10:20.0	50/52 (96)	1/283 (0)		10:10.0	48/48 (100)	0/306 (0)		11:02.0	24/24 (100)	23/23 (100)	1/302 (0)
	4	10:22.0	49/50 (98)	0/279 (0)		10:19.0	52/52 (100)	0/308 (0)		10:56.0	23/24 (96)	23/23 (100)	1/308 (0)
	5	10:25.0	45/48 (94)	1/279 (0)		10:21.0	50/50 (100)	1/325 (0)		10:54.0	24/24 (100)	22/23 (96)	1/298 (0)
	6	10:28.0	42/45 (93)	0/276 (0)		10:11.0	49/49 (100)	0/311 (0)		11:14.0	24/25 (96)	24/24 (100)	0/306 (0)
7	1	10:13.0	49/49 (100)	0/266 (0)	14	14:13.0	48/48 (100)	0/313 (0)	21	11:00.0	23/26 (88)	24/24 (100)	4/297 (1)
	2	10:09.0	50/50 (100)	0/260 (0)		18:52.0	50/50 (100)	2/326 (1)		11:27.0	24/26 (92)	26/26 (100)	1/290 (0)
	3	10:03.0	46/47 (98)	0/251 (0)		10:15.0	51/51 (100)	0/325 (0)		11:20.0	22/26 (85)	24/24 (100)	0/285 (0)
	4	10:03.0	50/50 (100)	0/251 (0)		10:07.0	49/49 (100)	1/322 (0)		11:05.0	20/25 (80)	24/24 (100)	3/285 (1)
	5	10:08.0	50/50 (100)	0/270 (0)		10:03.0	48/48 (100)	0/317 (0)		11:03.0	22/25 (88)	23/23 (100)	2/297 (1)
	6	10:08.0	48/48 (100)	0/270 (0)		10:09.0	48/48 (100)	4/315 (1)		11:07.0	22/25 (88)	25/25 (100)	3/292 (1)
Average		10:11.1	47.2/48.7 (96.9)	0.9/269.1 (0.2)		10:33.9	47.7/48.2 (99.0)	9.9/320.3 (2.3)		11:14.4	19.4/24.5 (78.9)	23.3/23.5 (99.1)	7.8/298.8 (1.8)
SD		00:13.9	2.3/1.8 (3.0)	2.3/10.9 (0.8)		01:31.3	2.9/2.6 (1.6)	35.7/9.0 (7.3)		00:21.0	5.2/1.3 (21.0)	1.0/1.0 (2.0)	37.4/14.7 (7.7)

Conventions are the same as in the Table 2 and 4.
